# The APC/C^FZY–1/Cdc20^ Complex Coordinates With OMA-1 to Regulate the Oocyte-to-Embryo Transition in *Caenorhabditis elegans*

**DOI:** 10.3389/fcell.2021.749654

**Published:** 2021-10-15

**Authors:** Yabing Hu, Xuewen Hu, Dongchen Li, Zhenzhen Du, Kun Shi, Chenxia He, Ying Zhang, Donglei Zhang

**Affiliations:** Department of Biochemistry and Molecular Biology, School of Basic Medicine, Tongji Medical College, Huazhong University of Science and Technology, Wuhan, China

**Keywords:** OMA-1, APC/C, ocyte-to-embryo transition, RNA binding protein, *C. elegans*

## Abstract

During oocyte maturation and the oocyte-to-embryo transition, key developmental regulators such as RNA-binding proteins coordinate translation of particular messenger RNA (mRNAs) and related developmental processes by binding to their cognate maternal mRNAs. In the nematode *Caenorhabditis elegans*, these processes are regulated by a set of CCCH zinc finger proteins. Oocyte maturation defective-1 (OMA-1) and OMA-2 are two functionally redundant CCCH zinc finger proteins that turnover rapidly during the first embryonic cell division. These turnovers are required for proper transition from oogenesis to embryogenesis. A gain-of-function mutant of OMA-1, *oma-1(zu405)*, stabilizes and delays degradation of OMA-1, resulting in delayed turnover and mis-segregation of other cell fate determinants, which eventually causes embryonic lethality. We performed a large-scale forward genetic screen to identify suppressors of the *oma-1(zu405)* mutant. We show here that multiple alleles affecting functions of various anaphase promoting complex/cyclosome (APC/C) subunits, including MAT-1, MAT-2, MAT-3, EMB-30, and FZY-1, suppress the gain-of-function mutant of OMA-1. Transcriptome analysis suggested that overall transcription in early embryos occurred after introducing mutations in APC/C genes into the *oma-1(zu405)* mutant. Mutations in APC/C genes prevent OMA-1 enrichment in P granules and correct delayed degradation of downstream cell fate determinants including pharynx and intestine in excess-1 (PIE-1), posterior segregation-1 (POS-1), muscle excess-3 (MEX-3), and maternal effect germ-cell defective-1 (MEG-1). We demonstrated that only the activator FZY-1, but not FZR-1, is incorporated in the APC/C complex to regulate the oocyte-to-embryo transition. Our findings suggested a genetic relationship linking the APC/C complex and OMA-1, and support a model in which the APC/C complex promotes P granule accumulation and modifies RNA binding of OMA-1 to regulate the oocyte-to-embryo transition process.

## Introduction

Early embryogenesis is a fundamental cellular process during development in metazoans. The specification of early cell fates and developmental patterns are usually determined at this stage. The process of early embryogenesis is governed by maternal mRNA and proteins loaded into eggs during oogenesis (Moore, 2005; Schier, 2007; Farley and Ryder, 2008). Maternal messenger RNA (mRNAs) are translationally repressed when loaded into eggs and their translations are regulated by a set of maternal loaded RNA-binding proteins (Schier, 2007; Rosario et al., 2017). During the oocyte-to-embryo transition, RNA-binding proteins coordinate translation of particular mRNAs and their corresponding developmental processes, such as anterior–posterior axis formation and cell fate specification (Newport and Kirschner, 1982; Edgar and Schubiger, 1986; Powell-Coffman et al., 1996; Oh and Houston, 2017). Shortly after, transcription from the zygotic genome begins and control of embryonic development is shifted from maternal to zygotic gene products. Meanwhile, primordial germ cells (PGCs) are specified during early embryogenesis. PGCs are kept transcriptionally quiescent until they need to proliferate, which also requires maternal supplied factors such as RNA-binding proteins (Forbes and Lehmann, 1998; Dansereau and Lasko, 2008).

**FIGURE 1:**
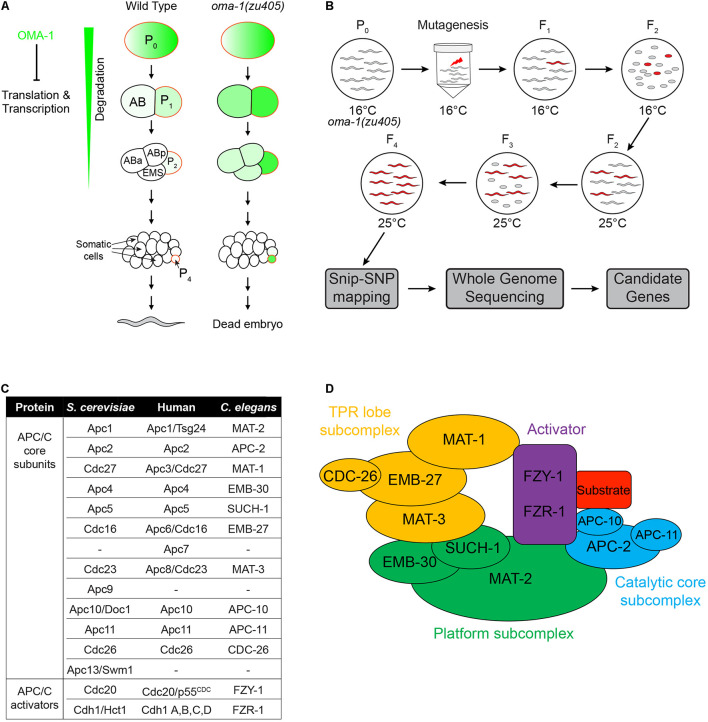
Schematic of the suppressor genetic screen to identify factors involved in OMA-1-regulated early embryogenesis in *C. elegans*. **(A)** Early embryonic development from a 1-cell to an ∼20-cell stage embryo in the wild type and *oma-1(zu405)* mutant, highlighting the expression of OMA-1 in P_0_ and P_1_ cells and delayed degradation in P_2_ and P_4_ cells. **(B)** Suppressor screen schematic. Synchronized *oma-1(zu405)* L4 animals were exposed to the mutagen, and phenotypes were scored in F_3_ and F_4_ at 25°C. Snip-SNP mapping and whole genome sequencing were performed to determine candidate genes. **(C)** Homologs of APC/C genes in *Saccharomyces cerevisiae*, humans, and *C. elegans*. It should be noted that not all homologs of APC/C genes in *S. cerevisiae* and humans are identified in *C. elegans*. **(D)** Schematic structure of the APC/C complex. The APC/C is generally divided into three subcomplexes and one activator.

The oocyte-to-embryo transition and early embryogenesis is rapid and dynamic in *Caenorhabditis elegans*, which provides a powerful model system to study these processes (Robertson and Lin, 2015). After fertilization, the *C. elegans* zygote undergoes several rounds of asymmetric divisions to produce particular early blastomeres that produce differentiated descendants and germ line cells. Specifically, during the first four rounds of asymmetric divisions, each division generates a somatic blastomere and a germline blastomere, named P_1_, P_2_, P_3_, and P_4_ (Figure 1A; Matova and Cooley, 2001; Rose and Gonczy, 2014). Later, P_4_ divides once to produce the PGCs, Z_2_, and Z_3_ (Wang and Seydoux, 2013). In order to ensure accurate completion of this process, maternal loaded mRNAs and RNA-binding proteins are needed to follow some special spatial and temporal localization patterns. More importantly, these RNA-binding proteins must have the ability to select their own targets from a large variety of maternal mRNAs.

Previous studies have identified a set of RNA-binding proteins that bind to specific mRNAs in different blastomere cells and at different developmental stages (Lee and Schedl, 2006). Among these RNA-binding proteins, oocyte maturation proteins OMA-1 and OMA-2, two closely related and functionally redundantly CCCH zinc finger proteins, play unique roles in oocyte maturation and the oocyte-to-embryo transition (Detwiler et al., 2001; Kaymak and Ryder, 2013). OMA-1/2 protein expression starts in developing oocytes and peaks in maturated oocytes and newly fertilized eggs. After the first embryonic division, OMA-1/2 proteins are rapidly degraded and are barely detectable in 4-cell stage embryos. Rapid degradation of OMA-1/2 proteins is required to avoid embryonic lethality (Lin, 2003). Degradation of oocyte maturation defective-1 (OMA-1) is regulated by DYRK Kinase MBK-2. During the oocyte-to-embryo transition, MBK-2 directly phosphorylates OMA-1 and promotes rapid turnover of OMA-1 (Shirayama et al., 2006). A gain of function mutation, P240L, in OMA-1 reduces MBK-2’s ability to phosphorylate OMA-1, resulting in increased protein stability and embryonic lethality (Shirayama et al., 2006). OMA-1/2 have been proposed to regulate the oocyte-to-embryo transition by targeting specific maternal mRNAs and repressing their translation (Lin, 2003). Previous studies have identified several targets of OMA-1/2, such as *zif-1* and *nos-2* (Jadhav et al., 2008; Guven-Ozkan et al., 2010). *nos-2* is a *nanos* homolog that is required for development of PGCs (Jadhav et al., 2008), while *zif-1* encodes a subunit of E3 ubiquitin ligase, which is required to restrict downstream cell fate determinants to proper localization through proteolysis (DeRenzo et al., 2003). One direct target of ZIF-1 is pharynx and intestine in excess-1 (PIE-1), another CCCH zinc finger protein, which is an essential germ cell fate determinant and it segregates with the germ cell lineage (DeRenzo et al., 2003). PIE-1 is accumulated in the nuclei of germline blastomeres to inhibit mRNA transcription. Hyper stabilized OMA-1 by the point mutation P240L causes delayed expression of ZIF-1 and abnormal distribution of PIE-1 from only being in germline blastomeres to being in somatic cells (Lin, 2003). Besides PIE-1, other cell fate determinants, including MEX-1, muscle excess-3 (MEX-3), MEX-5, and posterior segregation-1 (POS-1), which are also RNA-binding proteins and essential for embryogenesis, are incorrectly segregated and localized if OMA-1 is not properly degraded during the oocyte-to-embryo transition (Lin, 2003). OMA-1 and OMA-2 not only bind maternal mRNAs to repress translation, but also bind TATA box-binding protein (TBP)-associated factor TAF-4, a component that is essential for assembly of a functional TFIID and RNA polymerase II pre-initiation complex and to sequester TAF-4 in the cytoplasm in P_0_ and P_1_ germline blastomeres (Guven-Ozkan et al., 2008). MBK-2 directed phosphorylation promotes degradation of OMA-1 and allows TAF-4 to enter the nuclei of somatic cells to initiate zygotic transcription (Guven-Ozkan et al., 2008).

Although the key events of OMA-1/2 mediated oocyte maturation and oocyte-to-embryo transition are understood, the molecular mechanisms regulating OMA-1/2-mRNA binding, especially in the situation when OMA-1 is not properly degraded, are poorly understood. In this study, we designed a forward genetic screen to identify suppressors of the gain-of-function mutation P240L in OMA-1 (Lin, 2003). Using this unbiased genetic screen, we discovered that the anaphase promoting complex/cyclosome (APC/C) complex, especially when binding to activator Cdc20, participates in OMA-1 mediated oocyte maturation and the oocyte-to-embryo transition.

The APC/C complex is an E3 ubiquitin ligase that targets proteins for degradation during cell cycles, particularly during mitotic exit and the onset of anaphase (Harper et al., 2002; Peters, 2002, 2006; Satyanarayana and Kaldis, 2009; Pines, 2011). The APC/C complex is a large complex containing 11–13 subunits in different organisms, including a catalytic core of a Cullin subunit Apc2 and a Ring domain protein Apc11. The substrate recognition is specified by the activators Cdc20 and Cdh1 (Deshaies, 1999; Passmore, 2004; Pintard et al., 2004). The main substrate of the APC/C^Cdc20^ complex is securin, whose degradation leads to activation of separase and promotion of anaphase onset (Fang et al., 1999; Lara-Gonzalez et al., 2017), whereas the main substrate of APC/C^Cdh1^ is cyclin B, whose degradation leads to downregulation of CDK activity and is required for mitotic exit (Fang et al., 1998, 1999; Blanco et al., 2000; Listovsky et al., 2000; Lara-Gonzalez et al., 2017). All APC/C subunits are highly conserved from yeast to humans, and the molecular architecture of the APC/C complex has been elucidated recently (Dube et al., 2005; Passmore et al., 2005; Herzog et al., 2009; Uzunova et al., 2012; Primorac and Musacchio, 2013; Chang et al., 2014). Subunits of the APC/C complex can be grouped into three subcomplexes, including the platform, the catalytic core and the tetratricopeptide repeat (TPR) lobe (Figures 1C,D). Functions of APC/C during the oocyte-to-embryo transition have been studied previously, focusing on the release of meiosis arrest at the beginning of the oocyte-to-embryo transition (Marangos et al., 2007; Verlhac et al., 2010; Whitfield et al., 2013). Meiosis arrest happens during oocyte maturation in most animals, mainly because of the presence of cyclin B. The APC/C complex is activated upon fertilization and targets cyclin B for degradation to allow meiosis to proceed (Blanco et al., 2001; Pesin and Orr-Weaver, 2007; Swan and Schupbach, 2007; Radford et al., 2008). However, whether the APC/C complex plays other roles during oocyte maturation and the oocyte-to-embryo transition is still unclear.

In this study, we found that, although OMA-1 is not the direct target of the APC/C complex, the APC/C complex may participate in a pathway that regulates the association between OMA-1 and its cognate mRNA targets. We showed that mutations in multiple APC/C genes suppress embryonic lethality of the *oma-1(zu405)* strain, which has the gain-of-function mutation P240L in OMA-1 to impedes oocyte maturation. Transcriptome analysis of early embryos indicated gene expressions in *oma-1(zu405)* early embryos are similar to the wild type strain after introducing mutations in APC/C genes. We also showed that, although the APC/C complex does not regulate the degradation of OMA-1, it enhances P granule localization of OMA-1, where OMA-1 binds to its mRNA targets. This implies that the APC/C complex may modify the binding ability of OMA-1 to its cognate targets. Finally, proper segregation of some key cell fate determinants, including PIE-1, POS-1, MEX-3, and maternal effect germ-cell defective-1 (MEG-1), are corrected in the *oma-1(zu405)* mutant after introducing mutations in APC/C genes.

## Results

### A Suppressor Genetic Screen to Identify Factors Involved in OMA-1-Regulated Early Embryogenesis

In *oma-1(zu405)* embryos, the C blastomere is transformed to the ethyl methanesulfonate (EMS) blastomere fate at a restrictive temperature, resulting in temperature sensitive embryonic lethality (Figure 1A; Lin, 2003). We performed the suppressor screen to identify mutations rescuing embryonic lethality of the *oma-1(zu405)* strain. During the screen, F2 progenies of EMS/N-ethyl-N-nitrosourea (ENU) treated *oma-1(zu405)* worms were cultured at 25°C to determine whether F3 eggs were hatched or not. All viable F3 worms were collected for further study (Figure 1B). A total of 0.6 × 10^5^ F2s (i.e., 1.2 × 10^5^ genomes) were screened and 23 mutant strains were isolated. We used snip-SNPs as molecular markers to roughly map mutations in different chromosomes, then we performed whole genome sequencing (WGS) for *oma-1(zu405)* and all mutants, and analyzed the mutated genes with reference to the corresponding mapped chromosomes (Figure 1B). By comparing mutated genes in different strains, we identified three strains that had independent mutations in *mat-1*, two strains that had independent mutations in *mat-3*, and four strains that had independent mutations in *fzy-1*. Interestingly, MAT-1, MAT3, and FZY-1 all belong to subunits of the APC/C complex (Kitagawa et al., 2002; Shakes et al., 2003; Garbe et al., 2004). APC/C is a complex that has E3 ubiquitin ligase activity and plays a central role in the metaphase to anaphase transition and chromosome segregation steps during mitosis. To date, 12 subunits have been identified in yeast, 11 subunits have been identified in humans, and 10 subunits have been identified in *C. elegans* (Figure 1C; Furuta et al., 2000; Golden et al., 2000; Davis et al., 2002; Peters, 2002; Rappleye et al., 2002; Shakes et al., 2003; Passmore, 2004; Yeong, 2004; Pines, 2006). These subunits are organized into three functionally related subcomplexes: the platform (MAT-2/Apc1, EMB-30/Apc4, and SUCH-1/Apc5), the TPR lobe (MAT-1/Apc3, EMB-27/Apc6, MAT-3/Apc8, and CDC-26/Cdc26), and the catalytic core (APC-2/Apc2, APC-10/Apc10, and APC-11/Apc-11). There are also two activators, FZY-1/Cdc20 and FZR-1/Cdh1, that determine the target specificity of APC/C (Figure 1D).

### Variation of Mutations in Coding Genes of Subunits of the Anaphase Promoting Complex/Cyclosome Complex as Functional Genetic Alternations in Mutants From Ethyl Methanesulfonate Screens

**FIGURE 2:**
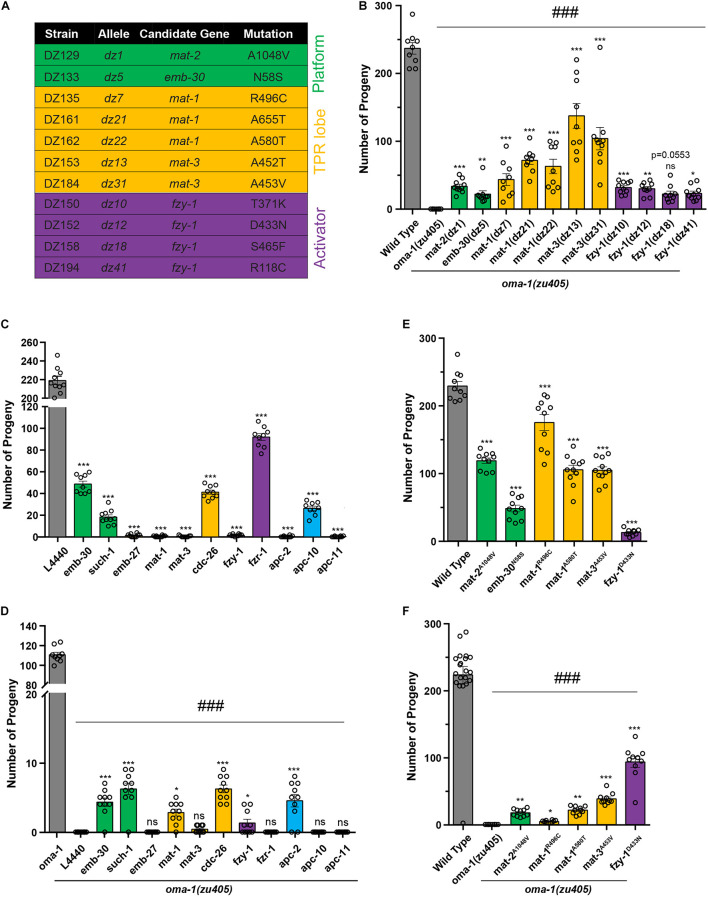
Mutations in APC/C genes suppressed lethality of the *oma-1(zu405)* mutant. **(A)** Summary of potential mutations in APC/C genes identified from the suppressor screen. Genes encoding for platform subunits, TRP lob subunits, and activators are highlighted in green, yellow, and purple, respectively. **(B)** Brood size of mutants identified from the suppressor screen of the *oma-1(zu405)* mutant. **(C)** Brood size of strains treated with RNAi of corresponding APC/C genes in the wild type N2. L4440 expressed an empty RNAi vector, which served as the control. **(D)** Brood size of strains treated with RNAi of corresponding APC/C genes in the *oma-1(zu405)* mutant. L4440 expressed an empty RNAi vector, which served as the control. **(E)** Brood size of strains contacting point mutations in APC/C genes generated by the CRISPR/Cas9 gene editing system. It should be noted that these point mutations were identified in mutants from the suppressor screen. **(F)** Brood size of *oma-1(zu405)* combined with corresponding point mutations in APC/C genes. The results are presented as the average ± S.D. with individual values plotted. *p < 0.05; **p < 0.01; ***, ###*p* < 0.001; ns: not significant. The *p*-values were calculated by 1-way ANOVA followed by Dunnett’s multiple comparisons. *N* = 10–15.

We next asked whether other genes coding APC/C subunits have mutations in these strains. Eventually we identified one strain that had a mutation in *mat-2* and one strain that had a mutation in *emb-30* (Figure 2A). All mutations we identified in APC/C genes are recessive, since only homozygous, but not heterozygous, mutants suppressed lethality of *oma-1(zu405)*.

To confirm whether these candidate genes are functional genes that suppress lethality of *oma-1(zu405)*, we performed two sets of experiments and investigated animal viability by scoring brood size: (1) RNAi treatment of candidate genes in *oma-1(zu405)*, and (2) regeneration of exactly the same mutations of candidate genes in *oma-1(zu405)* identified in EMS screens using the clustered regularly interspaced short palindromic repeats (CRISPR)/CRISPR-associated protein 9 (Cas9) gene editing system. We first scored brood size for all strains that contained the APC/C related alleles from our forward genetic suppressor screen (Figure 2B). *oma-1(zu405)* has a very tight phenotype of absolutely no progenies that can escape the lethality phenotype at 25°C (Lin, 2003). Although not all alleles restored the brood size of *oma-1(zu405)* to the wild type, they all suppressed lethality of *oma-1(zu405)* and restored the brood size from ∼20 to ∼140 offspring per animal. When wild type worms were treated with *mat-1*, *mat-2*, *mat-3*, *emb-30*, and *fzy-1* RNAi, we found that knockdown of *mat-1*, *mat-3*, and *fzy-1* caused lethality of wild type worms, while knockdown of *mat-2* and *emb-30* only had a mild effect on the viability of wild type animals (Figure 2C). Previous studies indicate that known alleles of these genes are either lethal or temperature sensitive lethal (Furuta et al., 2000; Golden et al., 2000; Kitagawa et al., 2002), suggesting RNAi of *mat-2* and *emb-30* did not completely deplete functional proteins. To assess RNAi efficiency, we measured mRNA levels of corresponding genes by quantitative real-time PCR (qRT-PCR), and found that, in RNAi treated strains, mRNA levels were knocked down to 15–40% of wild type RNA levels (Supplementary Figure 1A). In addition, we treated a strain expressing OMA-1::GFP (see below) with *oma-1* RNAi, and found that the OMA-1 protein level was reduced to ∼20% of the wild type protein level (Supplementary Figure 1B). We next scored brood size after treating the *oma-1(zu405)* strain with *mat-1*, *mat-2*, *mat-3*, *emb-30*, and *fzy-1* RNAi. We found all RNAi treatments restored fertility in *oma-1(zu405)* to very low levels. RNAi treatment of *emb-30* on *oma-1(zu405)* produced the largest brood size, which was only about 5 (Figure 2D).

Next, we reconstituted exact mutations in the APC/C genes identified in EMS screens by the CRISPR/Cas9 gene editing system. The mutations we regenerated included *mat-1^*R*496*C*^*, *mat-1^*A*580*T*^*, *mat-2^*A*1048*V*^*, *mat-3^*A*453*V*^*, *emb-30^*N*58*S*^*, and *fzy-1^*D*433*N*^* (Supplementary Figure 1C). We first generated these mutations in the wild type N2 background, and we found strains introduced by these mutations had relatively large brood sizes, from the smallest of 12 (*fzy-1^*D*433*N*^*) to the largest of more than 175 (*mat-1^*R*496*C*^*) (Figure 2E). Considering other known mutants of these genes are lethal, these data suggest that mutants we identified contain hypomorph alleles of APC/C genes. We crossed these CRISPR strains with *oma-1(zu405)* and scored brood sizes in double mutants. All regenerated mutations by CRISPR suppressed lethality of *oma-1(zu405)* to similar levels of EMS mutants of corresponding genes (Figure 2F). These results demonstrated that mutations in key APC/C subunits are the basis for the phenotypes observed in the mutants we isolated from the genetic screen.

It should be noted that the CRISPR generated *fzy-1^*D*433*N*^* mutant only had a very small brood size; however, the *fzy-1^*D*433*N*^;oma-1(zu405)* double mutant had a brood size of more than 90. This means that *fzy-1^*D*433*N*^* and *oma-1(zu405)* mutually suppress each other’s lethality phenotype, which further implied that OMA-1-regulated the embryogenesis pathway and this pathway and the APC/C complex have strong genetic interactions.

### Depletion Functions of Other Anaphase Promoting Complex/Cyclosome Subunits Repressed *oma-1(zu405)* Lethality

Since 10 subunits and two activators of APC/C have been identified in *C. elegans*, we asked whether other components of APC/C suppress lethality of *oma-1(zu405)*. We treated *oma-1(zu405)* with RNAi for *apc-2*, *such-1*, *emb-27*, *apc-10*, *apc-11*, *cdc-26*, and *fzr-1*, and then scored brood sizes. We found knockdown of *apc-2*, *such-1*, and *cdc-26* restored the brood size of *oma-1(zu405)* from 0 to around 6, which was very similar to RNAi treatment for APC/C genes identified by genetic screens, while knockdown of other genes, including *emb-27*, *apc-10*, *apc-11*, and *fzr-1*, did not suppress *oma-1(zu405)* (Figures 2C,D). Considering the observation that RNAi treatment of APC/C genes we identified by genetic screen only mildly suppress *oma-1(zu405)*, but hypomorph alleles of these genes efficiently restored viability of *oma-1(zu405)* (Figures 2C,D), we suspected that *apc-2*, *such-1*, and *cdc-26* could also have the same genetic interactions with *oma-1(zu405)*. RNAi of *emb-27* and *apc-11* caused complete lethality of wild type animals, indicating RNAi of these two genes were efficient. Although mRNA levels of *fzy-1* and *apc-10* were reduced to similar mRNAs levels of other APC/C genes after RNAi treatments (Supplementary Figure 1A), we cannot completely rule out that RNAi of *fzr-1* and *apc-10* depleted all function proteins because *fzr-1* RNAi and *apc-10* RNAi animals are not lethal. Among all genes that were investigated, *fzy-1* and *fzr-1* encoded the only two activators of the APC/C complex in *C. elegans*, which were homologs of yeast Cdc20 and Cdh1, respectively (Fay et al., 2002; Kitagawa et al., 2002). The main substrate of APC/C^Cdc20^ is securin, which inhibits separase and prevents chromosome segregation, and the main substrate of APC/C^Cdh1^ is cyclin B, which when removed can promote metaphase to anaphase during mitosis. It is interesting that only mutants of *fzy-1*, but not *fzr-1*, suppressed lethality of *oma-1(zu405)*, indicating the APC/C^Cdc20^ complex plays a particular role in the *oma-1*-regulated oocyte-to-zygote transition and oocyte maturation processes. Since among all mutants of APC/C genes, *fzy-1^*D*433*N*^* restored the brood size of *oma-1(zu405)* the best, we next used *fzy-1^*D*433*N*^* as the representative mutant of APC/C genes in the following studies.

### Depletion of Anaphase Promoting Complex/Cyclosome Subunits Partially Correct Embryonic Transcriptomes in the *oma-1(zu405)* Mutant

**FIGURE 3:**
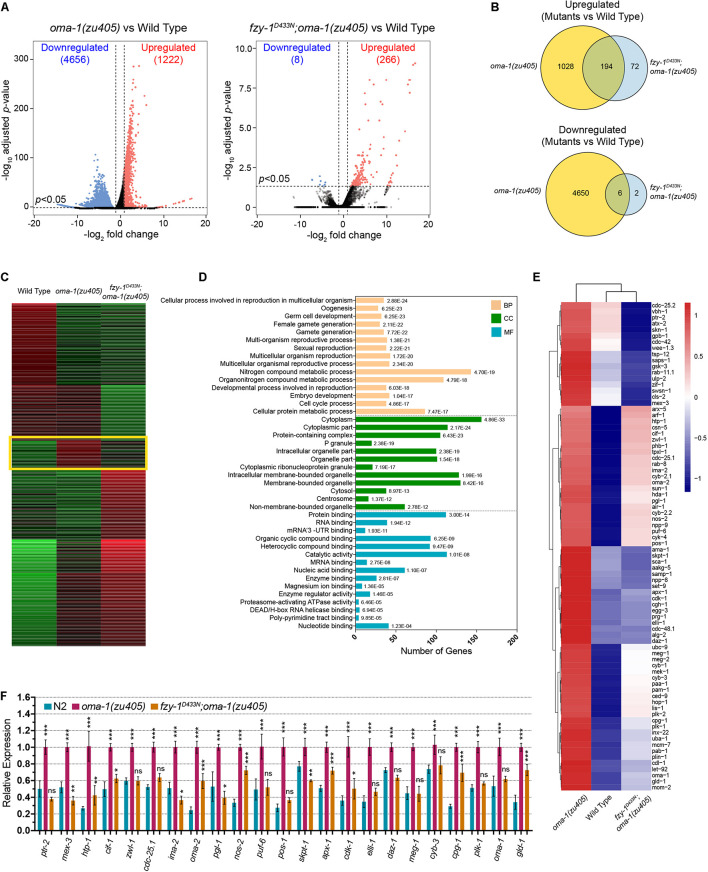
Comparative transcriptome analysis of *oma-1(zu405)*, *fzy-1^*D*433*N*^;oma-1(zu405)*, and the wild type. **(A)** Volcano plots of all differentially expressed genes detected in the mRNA-seq data in *oma-1(zu405)* (left) and *fzy-1^*D*433*N*^;oma-1(zu405)* (right), compared to the wild type. Criteria of deferential expression was | log_2_(fold change)| > 2, and Benjamini-Hochberg adjusted *p*-value < 0.05. **(B)** Venn diagrams depict the number of genes that are upregulated (or downregulated) only in *oma-1(zu405)* or *fzy-1^*D*433*N*^;oma-1(zu405)* or in both. **(C)** *K*-means cluster analysis of differentially expressed genes. The group of genes highlighted in the yellow rectangle show similar expression levels in *fzy-1^*D*433*N*^;oma-1(zu405)* and wild type strains. Expression levels were calculated as RPKM-normalized values. Each row represents a differentially expressed gene. **(D)** GO enrichment analysis of genes within the yellow rectangle in panel **(C)**. Category distribution of the 318 differentially expressed genes is shown for three GO domains: biological process (BP), molecular function (MF), and cellular component (CC). **(E)** The heatmap shows differential expression of 80 genes, which were assigned to the terms of germ cell development, embryo development, protein binding, and RNA binding in panel **(D)**. **(F)** Quantitative real-time PCR measurements of expression levels of selected 23 genes from (E) in the indicated strains. The results are presented as the average ± S.D. *p < 0.05; **p < 0.01; ***p < 0.001; ns: not significant. The *p*-values were calculated by 2-way ANOVA followed by Dunnett’s multiple comparisons.

To better characterize how mutations in APC/C complex suppress oma-1(zu405) lethality, we investigated transcriptome changes in *fzy-1^*D*433*N*^;oma-1(zu405)* double mutants versus the *oma-1(zu405)* single mutant. For this purpose, we isolated early embryos of corresponding strains, extracted total RNA, and performed mRNA sequencing (mRNA-seq) analysis. The isolated embryos were all from synchronized early young adults, and almost all were ∼30-cell or earlier stage embryos. We compared transcriptomes of *oma-1(zu405)* and *fzy-1^*D*433*N*^;oma-1(zu405)* to the wild type strain N2. Differentially expressed genes for each comparison were counted and identified by DESeq2. We identified 1,222 mRNAs that were upregulated and 4,656 mRNAs that were downregulated in the *oma-1(zu405)* mutant compared to the wild type N2 strain (| log_2_(fold change)| > 2, *p* < 0.05, Benjamini-Hochberg adjusted *p*-value). Based on the same criteria, mis-regulated expressions of mRNAs were largely corrected to normal levels, with only 266 mRNAs that were upregulated and eight mRNAs that were downregulated in the *fzy-1^*D*433*N*^;oma-1(zu405)* double mutant (Figure 3A). Venn diagrams showed that 192 mRNAs were commonly upregulated in both *oma-1(zu405)* and the *fzy-1^*D*433*N*^;oma-1(zu405)* mutants, while only six mRNAs were commonly downregulated (Figure 3B). These mRNA-seq data indicated mutations in APC/C genes can in general restore the overall disordered transcriptome in the *oma-1(zu405)* mutant to the wild type.

To better understand our mRNA-seq data, we used *k*-means clustering to assign gene expression levels into groups, and aimed to identify which group of genes were most affected when introducing *fzy-1^*D*433*N*^* into the *oma-1(zu405)* mutant. One group containing 318 genes was particularly notable, since in this group, expression levels of genes were similar in the wild type and *fzy-1^*D*433*N*^;oma-1(zu405)* mutant, implying expression of this group of genes is best representative of the overall gene expression levels (Figure 3C). We next performed Gene Ontology (GO) enrichment analysis for these 318 genes and determined the most significant terms in biological process (BP), cellular compartment (CC), and molecular function (MF). The terms were ordered from most enriched (up) to least enriched (bottom) in each category (Figure 3D). Interestingly, the terms assigned to biological processes with the most enrichment were involved in germ cells and embryo development; the terms assigned to CCs included protein containing complexes, P granules, and cytoplasmic ribonucleoprotein (RNP) granules; and the terms assigned into CCs were involved in protein and nucleic acid binding, especially mRNA and mRNA 3′UTR binding (Figure 3D). The data from the GO enrichment analysis further implicated mutations in APC/C genes promote normal embryonic development of the *oma-1(zu405)* strain, which may be caused by regulating cellular distributions of OMA-1 and its downstream cell fate determinants, including RNA and transcription factor-binding proteins.

We chose 80 genes that were assigned to the terms of germ cell development, embryo development, protein binding, and RNA binding and drew a heatmap of expression levels of these genes. These genes are among the genes with the most relevant functions during OMA-1-regulated embryogenesis and have the most dramatic differential expression levels, including *zif-1*, *pos-1*, *meg-1*, *meg-2*, and *skn-1*. Again, the heatmap showed that introducing *fzy-1^*D*433*N*^* could change expression levels of these genes in the *oma-1(zu405)* mutant to wild type (Figure 3E). Finally, we chose 23 genes from these 80 genes and performed qRT-PCR to evaluate expression levels. qRT-PCR data confirmed that changes in gene expression levels in *oma-1(zu405)*, *fzy-1^*D*433*N*^;oma-1(zu405)*, and wild type strains were consistent with mRNA-seq data (Figure 3F).

### Depletion of Anaphase Promoting Complex/Cyclosome Subunits Corrected Defects of the OMA-1 Distribution in the *oma-1(zu450)* Mutant

**FIGURE 4:**
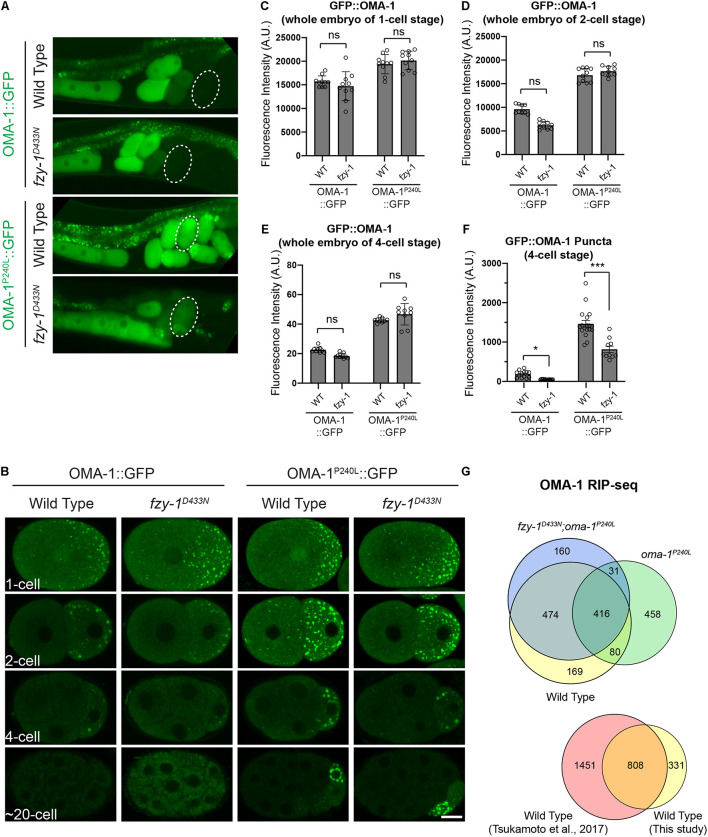
*fzy-1^*D*433*N*^* mutant affected the OMA-1 distribution in early embryos. **(A)** Fluorescent micrographs show the localization of OMA-1::GFP and OMA-1^P240L^::GFP in intact gonads in the wild type and *fzy-1^*D*433*N*^* mutant. 4-cell stage embryos are indicted by dotted white circles. **(B)** Fluorescent micrographs show the localization of OMA-1::GFP and OMA-1^P240L^::GFP in 1-cell, 2-cell, 4-cell, and ∼20-cell stage embryos in the wild type and *fzy-1^*D*433*N*^* mutant. Scale bar: 10 microns. **(C)** Quantification of overall OMA-1::GFP fluorescence in 1-cell stage embryos. **(D)** Quantification of overall OMA-1::GFP fluorescence in 2-cell stage embryos. **(E)** Quantification of overall OMA-1::GFP fluorescence in 4-cell stage embryos. **(F)** Quantification of OMA-1::GFP fluorescence enriched in granules in 4-cell stage embryos. The results are presented as the average ± S.D. with individual values plotted. *p < 0.05; ***p < 0.001. The *p*-values were calculated by 2-way ANOVA followed by Dunnett’s multiple comparisons. *N* = 8–12. **(G)** Above: Venn diagram showing the overlap between OMA-1- and OMA-1^P240L^-associated RNAs in wild type and *fzy-1^*D*433*N*^* mutant with a twofold enrichment cutoff. Bottom: Venn diagram showing the overlap between OMA-1-associated RNAs identified in this study and a previous study (Tsukamoto et al., 2017).

To investigate whether APC/C mutants suppress lethality of *oma-1(zu405)* by regulating cellular distribution of OMA-1, we created *in situ* knock-in strains expressing OMA-1::GFP and OMA-1^P240L^::GFP using the CRISPR/Cas9 gene editing system. Brood size of OMA-1::GFP strain was similar as the untagged strain either in wild type or *oma-2* mutant background, indicating tagging green fluorescent protein (GFP) into C-terminal OMA-1 did not affect OMA-1 function (Supplementary Figure 1D). As in previous studies, OMA-1::GFP accumulated in oocytes and very early embryos and started to be degraded after the first embryonic cell division and disappeared after 4-cell embryos (Figures 4A,B). OMA-1^P240L^::GFP fluorescence was still detected easily in 4-cell embryos and signals in P2 cells were stronger than those in somatic cells. OMA-1^P240L^::GFP fluorescence was eventually only detectable in P4 cells (Figures 4A,B).

Next, we introduced *fzy-1^*D*433*N*^* into OMA-1::GFP and OMA-1^P240L^::GFP transgenic strains to investigate whether OMA-1^P240L^::GFP is properly degraded in early embryos of the *fzy-1^*D*433*N*^* mutant. To rule out the possibility that introducing *fzy-1^*D*433*N*^* caused changes of overall OMA-1 protein levels, we compared OMA-1::GFP levels in wild type and *fzy-1^*D*433*N*^* embryos at the same developmental stage. By measuring fluorescent signals within whole embryos, we found that *fzy-1^*D*433*N*^* did not affect overall OMA-1 expression at any developmental stages we investigated (Figures 4C–E). We found *fzy-1^*D*433*N*^* mutants affected the distribution of wild type OMA-1::GFP in early embryos (Figures 4A,B). Especially in 2- and 4-cell embryos, OMA-1::GFP was less localized into the puncta-like structure P granules (Figure 4B and see below). Quantification in the 4-cell embryos showed reduced P granule localization was statistically significant (Figure 4F). In addition, OMA-1^P240L^::GFP was also altered when APC/C mutants were introduced. Although OMA-1^*P*240*L*^:GFP fluorescence was still easily detected in 2-, 4-, or 20-cell embryos, its accumulation in P granules was reduced compared to OMA-1^P240L^::GFP in the APC/C wild type cells (Figure 4B and see below).

**FIGURE 5:**
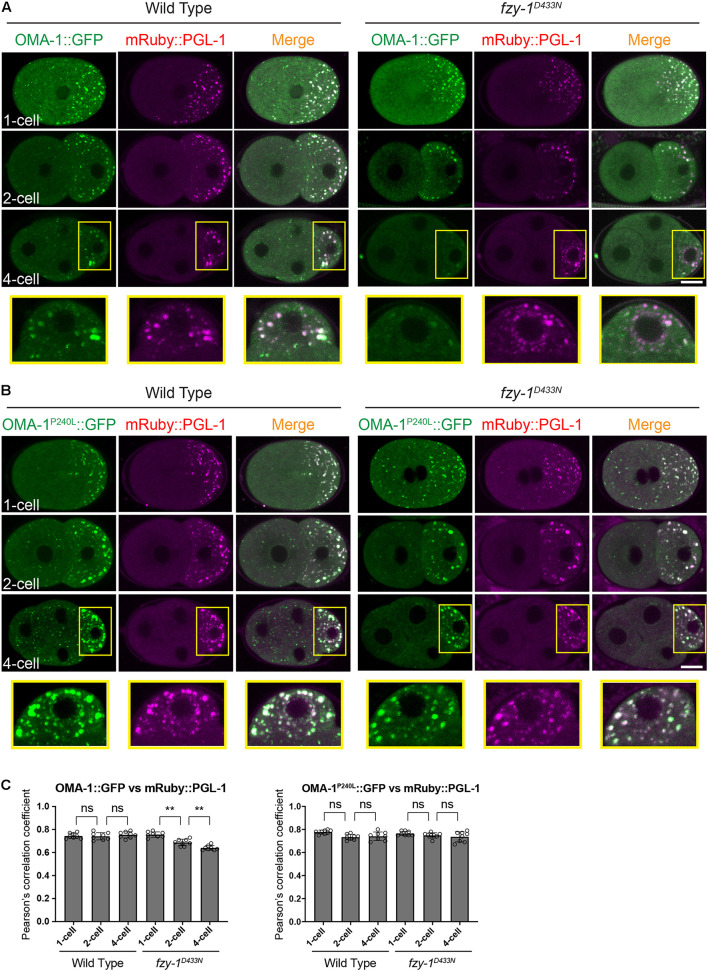
OMA-1::GFP and OMA-1^P240L^::GFP are localized to P granules in early embryos. **(A)** Fluorescent micrographs show the co-localization of OMA-1::GFP and mRuby::PGL-1 in P granules in 1-cell, 2-cell, and 4-cell stage embryos in the wild type and *fzy-1^*D*433*N*^* mutant. Regions within yellow rectangles are enlarged, turned 90 degrees counterclockwise, and displayed at the bottom. **(B)** Fluorescent micrographs show the co-localization of OMA-1^*P*240*L*^:GFP and mRuby::PGL-1 in P granules in 1-cell, 2-cell, and 4-cell stage embryos in the wild type and *fzy-1^*D*433*N*^* mutant. Regions within the yellow rectangles are enlarged, turned 90 degrees counterclockwise, and displayed at the bottom. Scale bar: 10 microns. **(C)** Pearson’s correlation coefficient to evaluate the extent of co-localization between OMA-1::GFP and mRuby::PGL-1. The results are presented as the average ± S.D. **p < 0.01; ns: not significant. The *p*-values were calculated by 2-way ANOVA followed by Dunnett’s multiple comparisons. *N* = 8.

Previous studies showed that OMA-1 accumulates at P granules in early embryos (Shimada et al., 2006). Considering untranslated maternal mRNAs accumulate at P granules during early embryogenesis (Noble et al., 2008), and OMA-1 binds to maternal mRNA to repress translation, it is reasonable to determine whether the APC/C complex regulates OMA-1 accumulation at P granules. We used mRuby::PGL-1 as the P granule marker, since PGL-1 is a typical component of P granules (Kawasaki et al., 1998). We first confirmed P granule accumulation of OMA-1 by showing that OMA-1::GFP and mRuby::PGL-1 were co-localized at P lineage cells in the wild type embryos (Figures 5A,C). By introducing the *fzy-1^*D*433*N*^* mutant, as we observed above, GFP:OMA-1 accumulated less to mRuby::PGL-1 labeled P granules, and Pearson’s correlation coefficients (PCCs) also indicated the reduction of co-localization between OMA-1::GFP and mRuby::PGL-1 in *fzy-1^*D*433*N*^* mutant (Figures 5A,C). Notably, highly enriched OMA-1^*P*240*L*^:GFP in P cells was also co-localized with mRuby::PGL-1, suggesting OMA-1^P240L^::GFP was indeed accumulated at P granules (Figure 5B). Surprisingly, we did not observe an obvious reduction of co-localization between OMA-1^*P*240*L*^:GFP and mRuby::PGL-1 in *fzy-1^*D*433*N*^* mutant (Figure 5C). It is possible that PGL-1 was also delocalized to P granules in OMA-1^P240L^::GFP genetic background. Another possibility is that mRuby::PGL-1 is overexpressed in this strain (Yang et al., 2014), which does not fully behave like the endogenous PGL-1 under certain circumstances. All the data above indicated APC/C mutants suppress *oma-1(zu405)* not by promoting degradation of OMA-1^P240L^ proteins, but possibly by restricting localization of OMA-1^P240L^ at P granules in early embryos. Less accumulation of OMA-1^P240L^ at P granules may reflect that OMA-1^P240L^ bound to different groups of mRNAs in APC/C mutants.

### Depletion of Anaphase Promoting Complex/Cyclosome Subunits Affected Association Between OMA-1 and mRNAs

To investigate whether APC/C complex regulate OMA-1-mRNA association, we immunopurified OMA-1::GFP by GFP nanobody and sequenced OMA-1-associated mRNAs in duplicate from young adults of *oma-1:gfp*, *oma-1^*P*240*L*^:gfp*, and *fzy-1^*D*433*N*^;oma-1^*P*240*L*^:gfp* strains. We also sequenced total RNAs in corresponding strains as input controls. OMA-1-associated mRNAs were identified using DESeq2 with a cutoff of | log2(fold change)| > 1 and Benjamini-Hochberg adjusted *p*-value < 0.05. Eventually, 1139 mRNAs were identified as OMA-1-associated mRNAs. To validate our analysis, we referred to published OMA-1 RIP-seq data (Tsukamoto et al., 2017), and found that 808/1139 (71%) mRNAs identified in our study were identified as OMA-1-assoicated mRNAs previously (Figure 4G), indicating our OMA-1 RIP-seq analysis was reliable. Based on the same criteria, 985 and 1081 mRNAs were identified as OMA-1^P240L^-associated mRNAs in the wild type and *fzy-1^*D*433*N*^* mutant, respectively. OMA-1 and OMA-1^P240L^ shared 496 mRNAs (44% of OMA-1- and 50% of OMA-1^P240L^-associated mRNAs), whereas OMA-1 and OMA-1^P240L^ in *fzy-1^*D*433*N*^* background shared 890 mRNAs (78% of OMA-1- and 82% of OMA-1^P240L^-assoicated mRNAs). Our data indicated that depletion of APC/C subunits affected OMA-1-RNA association, and turned mRNAs associated with OMA-1^P240L^ to be more similar to mRNAs associated with wild type OMA-1. It should be noted that numbers of mRNAs bound to OMA-1 and OMA-1^P240L^ were almost the same, implying that although mutation P240L altered OMA-1 binding affinity to untranslated mRNAs, the overall number of mRNAs bound to OMA-1 was largely unchanged. Developmental progresses during embryogenesis are largely determined by OMA-1 and OMA-1^P240L^ specifically associated RNAs.

### Depletion of Anaphase Promoting Complex/Cyclosome Subunits Correct Disrupted Distribution of Downstream Cell Fate Determinants of OMA-1, Including PIE-1, POS-1, MEX-3, and MEG-1

During embryogenesis, one of the mRNA targets OMA-1/2 binds to is *zif-1*. Binding of OMA-1/2 represses translation of *zif-1* (Guven-Ozkan et al., 2010). ZIF-1 encodes a subunit of E3 ligase whose direct substrate is PIE-1, an important cell fate determinant that segregates with the germ lineage for germ cell development during embryogenesis (Tenenhaus et al., 1998, 2001). Degradation of PIE-1 in somatic cells is delayed in *oma-1(zu405)* mutant embryos, possibly by delayed expression of ZIF-1. Degradation of several other maternal cell fate determinants, including MEX-1, MEX-3, MEX-5, POS-1, and MEG-1, was also delayed in *oma-1(zu405)* mutant embryos (Lin, 2003). To test whether APC/C mutants suppress delayed degradation of these cell fate determinants in the *oma-1(zu405)* strain, we investigated the distribution of CRISPR-mediated knock-in transgenic PIE-1::GFP, POS-1:GPF, and GFP::MEG-1, and a MosSCI-mediated single copy inserted transgenic GFP::MEX-3 in the *oma-1(zu405)* mutant and double mutants containing mutations of selected APC/C genes.

**FIGURE 6:**
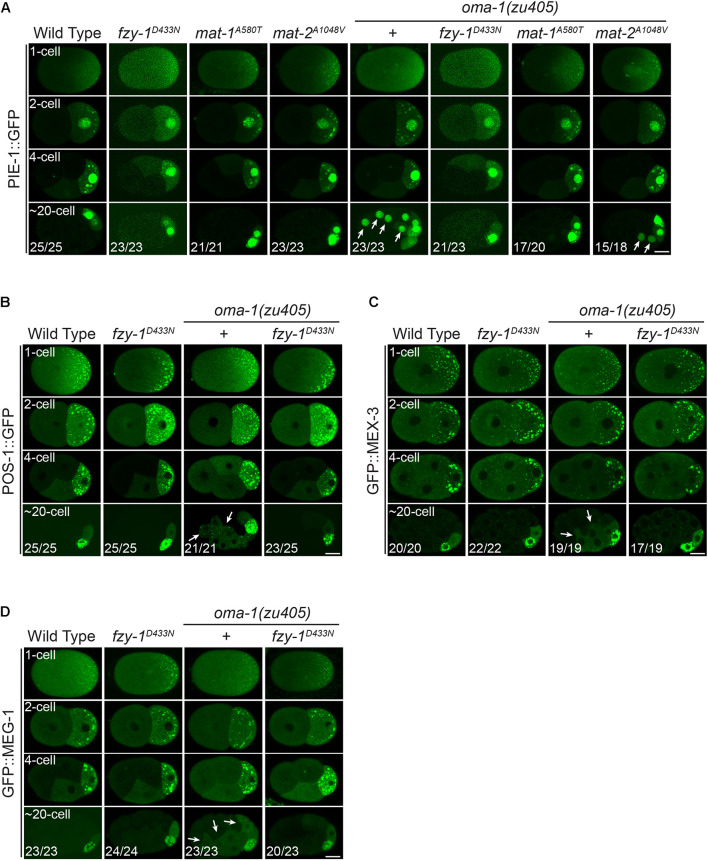
Mutants of APC/C genes correct the disrupted distribution of downstream cell fate determinants of OMA-1 in the *oma-1(zu405)* mutant. **(A)** Fluorescent micrographs show the localization of PIE-1::GFP in 1-cell, 2-cell, 4-cell, and ∼20-cell stage embryos in the wild type, *fzy-1^*D*433*N*^*, *mat-1^*A*580*T*^*, and *mat-2^*A*1048*V*^* mutants, with or without *oma-1(zu405)* in the background. Arrows indicate abnormal localization of PIE-1::GFP in somatic cells. **(B)** Fluorescent micrographs show the localization of POS-1::GFP in 1-cell, 2-cell, 4-cell, and ∼20-cell stage embryos in the wild type and *fzy-1^*D*433*N*^* mutants, with or without *oma-1(zu405)* in the background. Arrows indicate abnormal localization of POS-1::GFP in somatic cells. **(C)** Fluorescent micrographs show the localization of GFP::MEX-3 in 1-cell, 2-cell, 4-cell, and ∼20-cell stage embryos in the wild type and *fzy-1^*D*433*N*^* mutants, with or without *oma-1(zu405)* in the background. Arrows indicate abnormal localization of GFP::MEX-3 in somatic cells. **(D)** Fluorescent micrographs show the localization of GFP::MEG-1 in 1-cell, 2-cell, 4-cell, and ∼20-cell stage embryos in the wild type and *fzy-1^*D*433*N*^* mutants, with or without *oma-1(zu405)* in the background. Arrows indicate abnormal localization of GFP::MEG-1 in somatic cells. All phenotypes in 1-, 2-, and 4-cell embryos were 100% penetrant. Scale bar: 10 microns.

PIE-1 is a CCCH zinc finger protein that binds to the 3′UTR of maternal mRNAs to serve as an essential regulator of germ cell fate. Previous studies showed that PIE-1 is accumulated at P cells; i.e., germline precursor cells (Tenenhaus et al., 1998; Oldenbroek et al., 2013). We also observed that PIE-1:GFP asymmetrically localized at the posterior pole in 1-cell embryos so that PIE-1::GFP fluorescence in the anterior AB cell was weaker than that in the posterior P1 cell in 2-cell embryos. PIE-1::GFP further accumulated in P1, P2, and P4 cells as granule-like structures and in the nucleus, and at the same time, PIE-1:GFP in the somatic cells (i.e., non-P lineage cells) was rapidly degraded to levels below detection threshold (Figure 6A). On the other hand, in the *oma-1(zu405)* mutant, degradation of PIE-1::GFP in somatic cells was severely delayed, resulting in obvious nuclear located PIE-1::GFP signals in somatic cells at ∼20-cell embryos, although fluorescence in somatic cells was weaker than in P cells (Figure 6A). *fzy-1^*D*433*N*^*, *mat-1^*A*580*T*^*, or *mat-2^*A*1048*V*^* single mutants did not affect the dynamic distribution pattern of PIE-1::GFP in wild type animals. However, in the *oma-1(zu405)* mutant, *fzy-1^*D*433*N*^*, *mat-1^*A*580*T*^*, or *mat-2^*A*1048*V*^* mutants all promoted degradation of PIE-1::GFP to various levels. Specifically, somatic cell accumulation of PIE-1::GFP completely disappeared in *fzy-1^*D*433*N*^;oma-1(zu405)* and *mat-1^*A*580*T*^;oma-1(zu405)* double mutants, but only weak suppression of delayed degradation of PIE-1::GFP was shown in the *mat-2^*A*1048*V*^;oma-1(zu405)* double mutant since trace fluorescence signals were still detectable in some of the soma cells (Figure 6A). It should be noted that PIE-1::GFP only accumulated at the nucleus but not at cytoplasmic granules in somatic cells. The delayed degradation of nuclear PIE-1::GFP in the *oma-1(zu405)* mutant was also suppressed by APC/C mutants.

Similar to PIE-1, POS-1 is another CCCH zinc finger protein and cell fate determinant for germ cells, whose proper localization is disrupted in the *oma-1(zu405)* mutant (Lin, 2003; Farley et al., 2008). As with PIE-1::GFP, POS-1::GFP asymmetrically accumulates in granules at the posterior pole of 1-cell embryos, and eventually accumulates at P4 in ∼20-cell embryos after several rounds of asymmetric cell division (Figure 6B). In the *oma-1(zu405)* mutant, POS-1::GFP was still detectable in somatic cells rather than only in P4 in ∼20-cell embryos. Introduction of *fzy-1^*D*433*N*^* in *oma-1(zu405)* eliminated the remaining POS-1::GFP in somatic cells, recovering the defective distribution of POS-1::GFP to the wild type (Figure 6B).

We also investigated two other cell fate determinants; i.e., GFP::MEX-3 and GFP::MEG-1, which are essential for germ cell development. In the wild type, GFP::MEX-3 and GFP::MEG-1 only accumulated at P4 in ∼20-cell embryos after several rounds of asymmetric cell division. In the *oma-1(zu405)* mutant, GFP::MEX-3 and GFP::MEG-1 remained in the somatic cells, and most were cytoplasmic and a small portion accumulated at granules (Figures 6C,D). *fzy-1^*D*433*N*^* could fully remove the abnormal somatic remaining GFP::MEX-3 and GFP::MEG-1 in the *oma-1(zu405)* mutant. In the *fzy-1^*D*433*N*^;oma-1(zu405)* double mutant, localization of GFP::MEX-3 and GFP::MEG-1 behaved as in the wild type animals (Figures 6C,D).

All these results together indicated that disrupting APC/C functions could rescue delayed degradation of downstream cell fate determinants of OMA-1 caused by abnormal prolonged accumulation of OMA-1 protein during embryogenesis in the *oma-1(zu405)* mutant. This further suggested APC/C may regulate the binding ability of OMA-1 to RNAs or to proteins so that even OMA-1 is not degraded properly in the *oma-1(zu405)* mutant; it loses the ability to repress transcription or translation in early embryos. However, we cannot completely rule out the other possibility that APC/C indirectly regulates pathways that could bypass functions of OMA-1 to promote proper embryogenesis.

## Discussion

We report here that from a forward genetic suppressor screen for embryonic lethality of the *oma-1(zu405)* strain, mutations in five APC/C genes, represented by 11 alleles, were isolated (Figure 2A). We expanded our analysis to all APC/C subunits and found that subunits in all subcomplexes of APC/C participate in OMA-1 related oocyte maturation and the oocyte-to-embryo transition, including EMB-30/Apc4 and SUCH-1/Apc5 in the platform, MAT-1/Apc3, and CDC-26/Cdc26 in the TPR lobe, APC-2/Apc2 in the catalytic core, and activator FZY-1/Cdc20. Because depletion of APC/C functions usually cause lethality, we cannot rule out the role of EMB-27/Apc6, MAT-3/Apc8, and APC-11/Apc11 in these developmental processes (Figures 2C,D). One exception is that although depleting APC-10/Apc10 and FZR-1/Cdh1 did not cause lethality, *oma-1(zu405)* was not suppressed under these treatments. This indicated only the activator FZY-1/Cdc20, but not FZR-1/Cdh1, is incorporated in the APC/C complex to regulate early embryogenesis. Subunits from all subcomplexes participating suppression of *oma-1(zu405)* lethality indicates that the intact APC/C complex, but not any particular subunit or subcomplex, is needed to regulate oocyte maturation and the oocyte-to-embryo transition.

Many known alleles of APC/C genes are from screens for phenotypes of either abnormal embryogenesis (*emb* genes) or metaphase-to-anaphase transition defect (*mat* genes). Therefore, these alleles often cause lethality of animals. It should be noted the alleles identified in this study are viable and healthy, indicating they are hypomorph alleles of APC/C genes. Notably, nearly all mutations identified by our screens are located in protein-protein interaction domains of various subunits in APC/C complex. For example, the mutation of the platform subunit EMB-30, N58S, is in the WD40 domain; mutations of the TRP lob subunit MAT-1, R496C, A580T, and R655T, are all in the TRP repeats; mutations of another TRP lob subunit MAT-3, R452T, and A453V, are both in the TRP repeats; mutations of the co-activator subunit FZY-1, T371K, D433N, and S465F, are all in the WD40 domain. This provides a possible explanation that why those alleles are hypomorph. Mutations in theses alleles just affected amino acid residues within protein-protein interaction domains, which may change the overall stability of the APC/C complex and cause perturbation, but not complete depletion, of APC/C functions. APC-2 and APC-11 constitute the core catalytic module, and we did not identify any viable alleles of *apc-2* and *apc-11*, indicating that either any mutations in APC-2 or APC-11 would greatly affect functions of the APC/C complex, or our screen is not saturated. The hypomorph alleles are valuable reagents to study functions and molecular mechanisms of the APC/C complex, especially during the developmental stages of early embryogenesis.

RNA interference (RNAi) treatment of *mat-1*, *fzy-1*, or *apc-2* caused complete lethality in wild type animals, but not in the *oma-1(zu405)* strain, which suggests that, not only depleting the functions of these APC/C genes suppresses lethality of *oma-1(zu405)*, but *oma-1(zu405)* also suppresses lethality of mutations in these APC/C genes (Figures 2C,D). Another example is that the mutant with the hypomorph allele *fzy-1^*D*433*N*^* has a brood size of around 10, but *fzy-1^*D*433*N*^;oma-1(zu405)* has a brood size of more than 90 (Figures 2E,F). Since *oma-1(zu405)* is a gain-of-function allele, these mutual suppression effects may arise from accordant influences on oocyte maturation and the oocyte-to-embryo transition by the APC/C complex and OMA-1. Whether the APC/C complex regulates early embryogenesis through the OMA-1 related pathway needs more investigation, but our results provided some evidence to suggest that the APC/C complex may impair the RNA binding ability of OMA-1 (see below).

**FIGURE 7:**
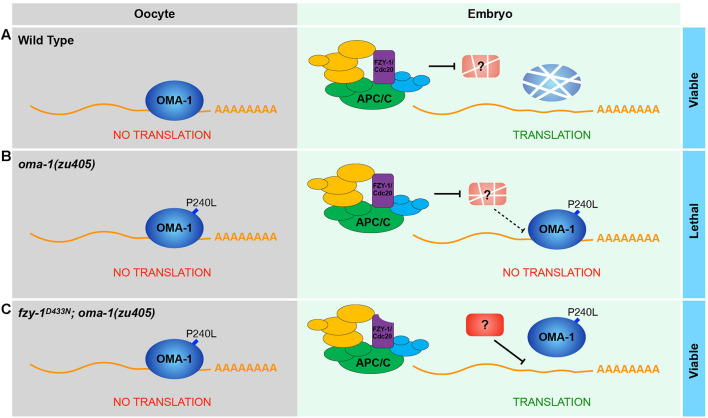
A proposed model to show how APC/C complex and OMA-1 are coordinated to regulate the oocyte-to-embryo transition. **(A)** In the wild type, OMA-1 binds to 3′UTR of its cognate mRNA to repress translation and undergoes rapid turnover in the oocyte-to-embryo transition. Maternal mRNAs can be translated normally in early embryos. **(B)** In the *oma-1(zu405)* mutant, the gain-of-function mutation P240L stabilizes OMA-1, causing OMA-1 continuously binds to its targets and repress their translation in early embryos, which eventually leads to embryonic lethality. **(C)** When the function of APC/C is perturbed (*fzy-1^*D*433*N*^* as an example), its substrates are not degraded. In this situation, P240L mutation still stabilizes OMA-1, however, the association between OMA-1^*P*240*L*^ and mRNA is weakened, which is probably regulated a potential APC/C substrate.

Our data showed that OMA-1 and OMA-1^P240L^ were less localized to P granules after introducing mutations in APC/C genes (Figures 4, 5). Previous studies suggested during early embryogenesis, untranslated maternal mRNAs are enriched in P granules and are released from P granules at specific developmental stages to exhibit corresponding functions (Spike et al., 2014; Lee et al., 2020; Parker et al., 2020; Aoki et al., 2021). Since we and others also found OMA-1 is enriched in P granules in 1-, 2-, and 4-cell stage embryos, considering OMA-1 binds to 3′UTR to repress translation of maternal mRNAs, it is reasonable to suggest that OMA-1 and its cognate mRNAs are associated with each other in P granules, and delocalization of OMA-1 from P granules indicates disassociation of OMA-1 from its cognate mRNAs (Figure 7). Our RIP-seq data also support this model. OMA-1 and OMA-1^P240L^ associate with different groups of mRNAs, and depletion of APC/C subunits turned mRNAs associated with OMA-1^P240L^ to be more similar to mRNAs associated with OMA-1. Due to technical difficulties, whole worms, rather than embryos, were used in this study to perform RIP-seq. Further validation of this model may need direct investigation of OMA-1-RNA bindings in defined developmental stage (i.e., 1-, 2-, and 4-cell embryos) during embryogenesis.

Because the APC/C complex is a E3 ubiquitin ligase that participates in proteasome mediated proteolysis, one key plan is to identify direct targets of the APC/C complex during developmental processes of oocyte maturation and the oocyte-to-embryo transition. Only the APC/C^Cdc20^ complex is involved in these processes, and the well-known target of the APC/C^Cdc20^ complex is securin. Degradation of securin leads to anaphase onset. If securin is the direct substrate of the APC/C^Cdc20^ complex, RNAi treatment of the *fzy-1^*D*433*N*^;oma-1(zu405)* strain with *ify-1*, the gene encoding securin homolog in *C. elegans* (Kitagawa et al., 2002), could switch phenotypes from the *fzy-1^*D*433*N*^;oma-1(zu405)* double mutant to the *oma-1(zu405)* single mutant. However, because RNAi of *ify-1* itself leads to embryos arresting at the one-cell stage [(Kitagawa et al., 2002) and data not shown], it is impossible to test whether securin is the direct substrate of the APC/C^Cdc20^ complex during early embryogenesis. It is possible that OMA-1 has a binding partner that prevents mRNA binding of OMA-1 and is the direct substrate of the APC/C^Cdc20^ complex. We performed OMA-1 immunoprecipitation followed by mass spectrometry. Again, depletion of nearly all potential OMA-1 binding partners caused lethality of animals, which excluded the possibility to direct test whether OMA-1 binding partners can serve as targets of the APC/C^Cdc20^ complex and their roles on OMA-1-RNA binding. Systematic analysis of binding partners and substrates of the APC/C^Cdc20^ complex may be helpful to identify which proteins are direct targets of the APC/C^Cdc20^ complex during OMA-1 regulated oocyte maturation and the oocyte-to-embryo transition.

## Materials and Methods

### *Caenorhabditis elegans* Strains

Animals were grown on standard nematode growth media (NGM) plates seeded with the *Escherichia coli* OP50 strain at 16, 20, or 25°C where indicated. Some of the *C. elegans* strains used in this study were purchased from the Caenorhabditis Genetics Center (CGC). Other strains were obtained through genetic screens or generated by the CRISPR/Cas9 gene editing system in this study. The strains that were used are listed in Supplementary Table 1.

### RNA Interference Treatment

RNA interference experiments were performed as described previously (Timmons and Fire, 1998). Monoclonal bacteria of HT115 (DE3) were inoculated and incubated overnight in LB medium supplemented with 100 μg/mL ampicillin at 37°C. Fresh cultures were seeded on NGM plates supplemented with 100 μg/mL ampicillin and 1 mM IPTG, and incubated at room temperature for 24 h before use. RNAi feeding bacterial strains were obtained from the *C. elegans* RNAi Collection (Ahringer; Source BioScience). L1–L3 stage animals were fed RNAi feed bacteria expressing corresponding double-stranded RNA at the normal culture temperature for 24 h, then shifted to 25°C for phenotype scoring. HT115 (DE3) expressing empty RNAi vector L4440 was used as the control.

### Forward Genetic Suppressor Screen

Forward genetic screens were performed as described (Brenner, 1974). EMS and ENU were combined to serve as mutagens in forward genetic screens (final concentration: 47 mM of EMS and 0.98 mM of ENU). Generally, *oma-1(zu405)* animals were treated with mutagens and mutants that could survive at 25°C were isolated. Synchronized L4 animals of the *oma-1(zu405)* strain were collected and incubated with EMS/ENU mutagens for 4 h at 16°C on a head-to-toe rotator. Animals were recovered and washed with M9 buffer (22 mM KH_2_PO_4_, 42 mM Na_2_HPO_4_, and 86 mM NaCl) five times and cultured at 16°C to adults. Animals were then bleached and synchronized F1s were collected. F1 progeny were cultured at 16°C with a density of around 300 animals per plate. After laying eggs, F1s were washed away with M9 buffer, and only F2 eggs were retained on plates. F2 was transferred to 25°C after reaching the L1 or L2 stage for further culture. Plates with significant amounts of F3 and F4 progenies were retained and mutants from those plates were isolated.

### Mapping and Cloning Candidate Genes Identified From the Suppressor Screen

We first sequenced the *oma-1* locus to exclude any mutation in *oma-1* coding sequences in each mutant strain. Mutants were crossed with the Hawaiian strain CB4856 and the Snip-SNP strategy was used to determine the genetic location of candidate genes, as described previously (Minevich et al., 2012). Next, genomic DNA was extracted from the mutants and *oma-1(zu405)* strain, and WGS was performed. WGS data from mutants and *oma-1(zu405)* were compared using homemade scripts. Combining the methods described above, candidate genes were determined. Reconstruction of candidate mutations were generated by the CRISPR/Cas9 gene editing system to eventually confirm the candidate genes.

### Brood Size Analysis

Single hermaphrodite L4s (P0s) were placed onto individual freshly seeded NGM plates and allowed to grow for 24 h at 25°C. P0 adults were transferred to new NGM plates every 24 h until they no longer laid eggs. All the F1 progeny on each plate were counted. The brood size of each P0 animal was the total sum of F1s for all plates where the P0 animal laid eggs. 10–15 P0 animals were used to calculated the brood size for each strain. Data were analyzed by 1-way ANOVA followed by Dunnett’s multiple comparisons.

### CRISPR/Cas9 Gene Editing

Single guide RNAs (sgRNAs) were designed with the online tool CHOPCHOP.^1^ sgRNA sequences used in this study are listed in Supplementary Table 2. Two strategies were used to obtain sgRNA. (1) The DNA sequences encoding sgRNAs were cloned into pDD162 by overlapping polymerase chain reaction (PCR) using appropriate primers and pDD162 as the PCR template (Dickinson et al., 2013). Overlapping PCR products were inserted into pDD162 linearized with *Spe*I/*Bsr*BI digestion with a ClonExpress Ultra One Step Cloning Kit (Vazyme #C115-01). (2) sgRNAs were synthesized and purified *in vitro* with a HiScribe Quick T7 High Yield RNA Synthesis Kit (New England Biolabs #E2050).

To generate the donor construct for *gfp* knock-in into the *oma-1* locus, 500 bp upstream and downstream DNA of the *oma-1* 3′UTR and GFP coding sequences were amplified by PCR using N2 genomic DNA or plasmids containing GFP coding sequences as templates. Overlapping PCR fragments were linearized with *Hin*dIII/*Kpn*I digestion with a ClonExpress Ultra One Step Cloning Kit (Vazyme #C115-01) and inserted into pUC19. Single-stranded oligonucleotides (ssODN) with 30 bp upstream and 30 bp downstream homology sequences and desired mutations were used as donors to introduce point mutations.

CRISPR experiments were conducted with co-CRISPR or Cas9 RNP strategies (Dokshin et al., 2018). For the co-CRISPR strategy, DNA mixtures were introduced into the germline of *C. elegans* young adults by microinjection. Final concentrations of plasmids in the injection mixtures were as follows: 50 ng/mL of pCCM935 *unc-22* sgRNA, 50 ng/mL of pDD162 Cas9 + sgRNA, 30 ng/mL of pRF4 *rol-6 (dm)* and 50 ng/mL of single-stranded DNA (ssDNA) donor oligo (or 50 ng/mL of plasmid-based donor). F1 twitchers or rollers were isolated, followed by genotyping of the desired mutations. When applicable, transgenic strains were outcrossed to remove *unc-22* mutations. For the RNP strategy, final concentrations of injection components were as follows: 250 ng/mL of Alt-R Cas9 protein (IDT), 100 ng/μL of Alt-R tracrRNA (IDT), and 56 ng/μL of customized Alt-R crRNA (IDT) or 200 ng/μL of customized Alt-R sgRNA (IDT). All components were mixed and incubated at 37°C for 10 min, then 110 ng/μL of ssDNA donor oligo or 200 ng/μL of dsDNA donor and 40 ng/μL of pRF4 *rol-6 (dm)* were added. Injection mixtures were introduced into the germline of *C. elegans* young adults by microinjection. The F1 rollers were isolated and genotyped to identify the desired mutations.

### Fluorescence Microscopy

GFP- and mRuby-tagged fluorescent proteins were visualized in living nematodes or dissected embryos by mounting young adult animals on 2% agarose pads with M9 buffer (22 mM KH_2_PO_4_, 42 mM Na_2_HPO_4_, and 86 mM NaCl) with 10–50 mM levamisole, or mounting one-cell embryos on 2% agarose pads by dissecting gravid hermaphrodites into egg salt buffer (5 mM HEPES pH = 7.4, 118 mM NaCl, 40 mM KCl, 3.4 mM MgCl_2_, and CaCl_2_ 3.4 mM). Fluorescent images were captured using a Zeiss LSM800 confocal microscope with a Plan-Apochromat 63X/1.4 Oil DIC M27 objective.

The quantification of fluorescent puncta (i.e., OMA-1::GFP and OMA-1^P240L^::GFP) was performed using ImageJ. Maximum intensity projections of z-series were obtained. Image thresholds were set manually and fluorescent puncta were selected. Integrated intensity of all puncta in a single embryo was measured and summed together. Images of 8–12 embryos were collected and quantified. Data were analyzed by 2-way ANOVA followed by Dunnett’s multiple comparisons.

The quantification of overall fluorescence of OMA-1::GFP and OMA-1^P240L^::GFP in embryos or germlines was performed using ImageJ. Maximum intensity projections of z-series were obtained. Region of interest (ROIs) was selected and integrated intensity of all pixels within an ROI was measured. Images of 8–12 embryos or germlines were collected and quantified. For quantification of OMA-1::GFP and OMA-1^P240L^::GFP in embryos, data were analyzed by 2-way ANOVA followed by Dunnett’s multiple comparisons. For quantification of OMA-1::GFP in germlines, data were analyzed by student’s *t*-test.

For quantitative co-localization analysis, all image manipulations were performed with ImageJ using the Coloc 2 plugin. PCCs were calculated. Data were analyzed by 1-way ANOVA followed by Dunnett’s multiple comparisons. *N* = 8 for each strains.

### Transcriptome Analysis by mRNA-seq

Synchronized L1 animals were cultured at 25°C until they grew into early young adults, which were then collected and washed with M9 buffer (22 mM KH_2_PO_4_, 42 mM Na_2_HPO_4_, and 86 mM NaCl) several times. Early embryos were obtained by bleaching and washing with M9 buffer five times and ice water for the last time. Total RNA was extracted using the standard method with TRIzol reagent (Invitrogen), and an mRNA library was generated for high throughput sequencing using the VAHTS Universal V8 RNA-seq Library Prep Kit for Illumina (Vazyme #NR605-01). Sequencing reads were generated with an Illumina NovaSeq 6000 system. All trimmed reads were aligned to the reference genome using HISAT2 (Kim et al., 2019). The read counts mapped to each transcript were calculated using HTSeq (Anders et al., 2015), and then normalized to reads per kilobases per million reads (RPKM). The results were used for the analysis of differentially expressed genes. All differentially expressed genes were identified using DESeq2 (Love et al., 2014) with a cutoff of | log_2_(fold change)| > 2 and Benjamini-Hochberg adjusted *p*-value < 0.05. *k*-means clustering analysis of differentially expressed genes was performed using iDEP 0.93^2^ (Ge et al., 2018). GO enrichment analysis of differentially expressed genes was performed with the GO Enrichment Analysis tool at https://www.omicshare.com/tools/.

### RNA Immunoprecipitation Sequencing

A total of 100,000 synchronized young adult animals were frozen in liquid nitrogen and stored at −80°C. Pellets were resuspended in equal volumes of immunoprecipitation buffer [20 mM Tris–HCl pH 7.5, 150 mM NaCl, 2.5 mM MgCl_2_, 0.5% NP-40, 80 U ml^–1^ RNase Inhibitor (Thermo), 1 mM dithiothreitol, and protease inhibitor cocktail without EDTA (Promega)], and grinded in a glass grinder for 8–10 times. The whole grinding process should not exceed 10 min. Lysates were clarified by spinning down at 15000 rpm, 4°C, for 15 min. Supernatants were incubated with the GFP-Trap magnetic agarose beads (ChromoTek) at 4°C for 1 h. Beads were washed with IP wash buffer (20 mM Tris–HCl pH 7.5, 150 mM NaCl, 2.5 mM MgCl_2_, 0.5% NP-40, and 1 mM dithiothreitol) six times, and then resuspended in TBS buffer for RNA extraction. Total RNA was extracted using the standard method with TRIzol reagent (Invitrogen). The mRNA library was generated, sequenced and analyzed as transcriptome analysis by mRNA-seq in this study described above to get RPKM values. RPKM values from IP samples and input samples were used for defining OMA-1-associated mRNAs by DESeq2 (Love et al., 2014) with a cutoff of | log_2_(fold change)| > 1 and Benjamini-Hochberg adjusted *p*-value < 0.05.

### Quantitative Real-Time PCR

RNA was extracted from the early embryos as described above. cDNA was then synthesized using HiScript II Q Select RT SuperMix for qPCR with gDNA wiper (Vazyme #R233-01) according to the manufacturer’s instructions. Real-time PCR was performed on a CFX Connect Thermal Cycler (Bio-Rad) with ChamQ SYBR qPCR Master Mix (Vazyme # Q311-02). Amplification was performed with a two-step reaction at 95°C for 3 min and 40 cycles at 95°C for 15 s and at 60°C for 30 s. A 20 μL PCR mixture included 10 μL ChamQ SYBR qPCR Master Mix, 2 μL primer mix (10 μM each), 3 μL diluted template cDNA, and 5 μL deionized distilled water. The relative fold changes in related genes were normalized to expression levels of actin, and analyzed by 2-way ANOVA followed by Dunnett’s multiple comparisons. Each experiment was repeated four times. The PCR primers used in this study are listed in Supplementary Table 2.

## Data Availability Statement

The datasets presented in this study can be found in online repositories. The names of the repository/repositories and accession number(s) can be found below: https://www.ncbi.nlm.nih.gov/geo/query/acc.cgi?acc=GSE181115.

## Author Contributions

YH carried out the majority of experiments and data analysis. XH conducted the bioinformatics analysis. DL conducted some of the crosses and genotyping. YH, ZD, KS, and CH performed the genetic screens. YH and ZD cloned mutated genes. DZ designed the study and wrote the manuscript. YZ and DZ supervised the work. All authors contributed to manuscript revision, read, and approved the submitted version.

## Conflict of Interest

The authors declare that the research was conducted in the absence of any commercial or financial relationships that could be construed as a potential conflict of interest.

## Publisher’s Note

All claims expressed in this article are solely those of the authors and do not necessarily represent those of their affiliated organizations, or those of the publisher, the editors and the reviewers. Any product that may be evaluated in this article, or claim that may be made by its manufacturer, is not guaranteed or endorsed by the publisher.

## Footnotes

1
https://chopchop.cbu.uib.no/?tdsourcetag=s_pcqq_aiomsg

2
http://bioinformatics.sdstate.edu/idep/

## Supplementary Material

Supplementary Figure 1 **(A)** Quantitative real-time PCR measurements of expression levels of indicated APC/C genes in RNAi treated strains. The results are presented as the average ± S.D. ^∗∗∗^*p* < 0.001; ns: not significant. The *p*-values were calculated by 2-way ANOVA followed by Dunnett’s multiple comparisons. **(B)** Fluorescent micrographs show OMA-1::GFP in germlines in control and *oma-1* RNAi treated strains to demonstrate *oma-1* RNAi efficiency. Quantification of overall OMA-1::GFP fluorescence in germlines is presented as the average ± S.D. with individual values plotted. ^∗∗∗^*p* < 0.001. The *p*-values were calculated by student’s *t*-test. *N* = 8–12. **(C)** Validation of mutations generated by CRISPR/Cas9 gene editing system by Sanger Sequencing. Wild type sequences are shown at the top and mutated sequences are shown at the bottom for each mutation. Red box, missense mutations causing desired amino acid residue change; blue box, silent mutations for genotyping. **(D)** Brood size of *oma-1(ne3800)* and *oma-1:gfp* strains in wild type and *oma-2* RNAi background. The results are presented as the average ± S.D. with individual values plotted. ^∗∗∗^*p* < 0.001; ns: not significant. The *p*-values were calculated by 1-way ANOVA followed by Dunnett’s multiple comparisons. *N* = 10–15.

## References

[B1] Anders S. Pyl P. T. Huber W. (2015). HTSeq–a Python framework to work with high-throughput sequencing data. *Bioinformatics* 31 166–169. 10.1093/bioinformatics/btu638 25260700 PMC4287950

[B2] Aoki S. T. Lynch T. R. Crittenden S. L. Bingman C. A. Wickens M. Kimble J. (2021). C. elegans germ granules require both assembly and localized regulators for mRNA repression. *Nat. Commun.* 12:996. 10.1038/s41467-021-21278-1 33579952 PMC7881195

[B3] Blanco M. A. Pelloquin L. Moreno S. (2001). Fission yeast mfr1 activates APC and coordinates meiotic nuclear division with sporulation. *J. Cell Sci.* 114 (Pt 11) 2135–2143. 11493649 10.1242/jcs.114.11.2135

[B4] Blanco M. A. Sanchez-Diaz A. de Prada J. M. Moreno S. (2000). APC(ste9/srw1) promotes degradation of mitotic cyclins in G(1) and is inhibited by cdc2 phosphorylation. *EMBO J.* 19 3945–3955. 10.1093/emboj/19.15.3945 10921876 PMC306614

[B5] Brenner S. (1974). The genetics of *Caenorhabditis elegans*. *Genetics* 77 71–94. 4366476 10.1093/genetics/77.1.71 PMC1213120

[B6] Chang L. F. Zhang Z. Yang J. McLaughlin S. H. Barford D. (2014). Molecular architecture and mechanism of the anaphase-promoting complex. *Nature* 513 388–393. 10.1038/nature13543 25043029 PMC4456660

[B7] Dansereau D. A. Lasko P. (2008). The development of germline stem cells in *Drosophila*. *Methods Mol. Biol.* 450 3–26. 10.1007/978-1-60327-214-8_1 18370048 PMC2729445

[B8] Davis E. S. Wille L. Chestnut B. A. Sadler P. L. Shakes D. C. Golden A. (2002). Multiple subunits of the *Caenorhabditis elegans* anaphase-promoting complex are required for chromosome segregation during meiosis I. *Genetics* 160 805–813. 11861581 10.1093/genetics/160.2.805 PMC1461975

[B9] DeRenzo C. Reese K. J. Seydoux G. (2003). Exclusion of germ plasm proteins from somatic lineages by cullin-dependent degradation. *Nature* 424 685–689. 10.1038/nature01887 12894212 PMC1892537

[B10] Deshaies R. J. (1999). SCF and cullin/ring H2-based ubiquitin ligases. *Annu. Rev. Cell Dev. Biol.* 15 435–467. 10.1146/annurev.cellbio.15.1.435 10611969

[B11] Detwiler M. R. Reuben M. Li X. Rogers E. Lin R. (2001). Two zinc finger proteins, OMA-1 and OMA-2, are redundantly required for oocyte maturation in *C. elegans*. *Dev. Cell* 1 187–199. 10.1016/s1534-5807(01)00026-0 11702779

[B12] Dickinson D. J. Ward J. D. Reiner D. J. Goldstein B. (2013). Engineering the *Caenorhabditis elegans* genome using Cas9-triggered homologous recombination. *Nat. Methods* 10 1028–1034. 10.1038/nmeth.2641 23995389 PMC3905680

[B13] Dokshin G. A. Ghanta K. S. Piscopo K. M. Mello C. C. (2018). Robust genome editing with short single-stranded and long, partially single-stranded DNA donors in *Caenorhabditis elegans*. *Genetics* 210 781–787. 10.1534/genetics.118.301532 30213854 PMC6218216

[B14] Dube P. Herzog F. Gieffers C. Sander B. Riedel D. Muller S. A. (2005). Localization of the coactivator Cdh1 and the cullin subunit Apc2 in a cryo-electron microscopy model of vertebrate APC/C. *Mol. Cell* 20 867–879. 10.1016/j.molcel.2005.11.008 16364912

[B15] Edgar B. A. Schubiger G. (1986). Parameters controlling transcriptional activation during early *Drosophila* development. *Cell* 44 871–877. 10.1016/0092-8674(86)90009-7 2420468

[B16] Fang G. Yu H. Kirschner M. W. (1998). Direct binding of CDC20 protein family members activates the anaphase-promoting complex in mitosis and G1. *Mol. Cell* 2 163–171. 10.1016/s1097-2765(00)80126-4 9734353

[B17] Fang G. Yu H. Kirschner M. W. (1999). Control of mitotic transitions by the anaphase-promoting complex. *Philos. Trans. R. Soc. Lond. B Biol. Sci.* 354 1583–1590. 10.1098/rstb.1999.0502 10582244 PMC1692672

[B18] Farley B. M. Pagano J. M. Ryder S. P. (2008). RNA target specificity of the embryonic cell fate determinant POS-1. *RNA* 14 2685–2697. 10.1261/rna.1256708 18952820 PMC2590972

[B19] Farley B. M. Ryder S. P. (2008). Regulation of maternal mRNAs in early development. *Crit. Rev. Biochem. Mol. Biol.* 43 135–162. 10.1080/10409230801921338 18365862

[B20] Fay D. S. Keenan S. Han M. (2002). fzr-1 and lin-35/Rb function redundantly to control cell proliferation in *C. elegans* as revealed by a nonbiased synthetic screen. *Genes Dev.* 16 503–517. 10.1101/gad.952302 11850412 PMC155341

[B21] Forbes A. Lehmann R. (1998). Nanos and pumilio have critical roles in the development and function of *Drosophila* germline stem cells. *Development* 125 679–690. 9435288 10.1242/dev.125.4.679

[B22] Furuta T. Tuck S. Kirchner J. Koch B. Auty R. Kitagawa R. (2000). EMB-30: an APC4 homologue required for metaphase-to-anaphase transitions during meiosis and mitosis in *Caenorhabditis elegans*. *Mol. Biol. Cell* 11 1401–1419. 10.1091/mbc.11.4.1401 10749938 PMC14855

[B23] Garbe D. Doto J. B. Sundaram M. V. (2004). *Caenorhabditis elegans* lin-35/Rb, efl-1/E2F and other synthetic multivulva genes negatively regulate the anaphase-promoting complex gene mat-3/APC8. *Genetics* 167 663–672. 10.1534/genetics.103.026021 15238519 PMC1470888

[B24] Ge S. X. Son E. W. Yao R. (2018). iDEP: an integrated web application for differential expression and pathway analysis of RNA-Seq data. *BMC Bioinformatics* 19:534. 10.1186/s12859-018-2486-6 30567491 PMC6299935

[B25] Golden A. Sadler P. L. Wallenfang M. R. Schumacher J. M. Hamill D. R. Bates G. (2000). Metaphase to anaphase (mat) transition-defective mutants in *Caenorhabditis elegans*. *J. Cell Biol.* 151 1469–1482. 10.1083/jcb.151.7.1469 11134076 PMC2150685

[B26] Guven-Ozkan T. Nishi Y. Robertson S. M. Lin R. (2008). Global transcriptional repression in *C. elegans* germline precursors by regulated sequestration of TAF-4. *Cell* 135 149–160. 10.1016/j.cell.2008.07.040 18854162 PMC2652481

[B27] Guven-Ozkan T. Robertson S. M. Nishi Y. Lin R. (2010). zif-1 translational repression defines a second, mutually exclusive OMA function in germline transcriptional repression. *Development* 137 3373–3382. 10.1242/dev.055327 20826530 PMC2947753

[B28] Harper J. W. Burton J. L. Solomon M. J. (2002). The anaphase-promoting complex: it’s not just for mitosis any more. *Genes Dev.* 16 2179–2206. 10.1101/gad.1013102 12208841

[B29] Herzog F. Primorac I. Dube P. Lenart P. Sander B. Mechtler K. (2009). Structure of the anaphase-promoting complex/cyclosome interacting with a mitotic checkpoint complex. *Science* 323 1477–1481. 10.1126/science.1163300 19286556 PMC2989460

[B30] Jadhav S. Rana M. Subramaniam K. (2008). Multiple maternal proteins coordinate to restrict the translation of *C. elegans* nanos-2 to primordial germ cells. *Development* 135 1803–1812. 10.1242/dev.013656 18417623 PMC2573031

[B31] Kawasaki I. Shim Y. H. Kirchner J. Kaminker J. Wood W. B. Strome S. (1998). PGL-1, a predicted RNA-binding component of germ granules, is essential for fertility in *C. elegans*. *Cell* 94 635–645. 10.1016/s0092-8674(00)81605-0 9741628

[B32] Kaymak E. Ryder S. P. (2013). RNA recognition by the *Caenorhabditis elegans* oocyte maturation determinant OMA-1. *J. Biol. Chem.* 288 30463–30472. 10.1074/jbc.M113.496547 24014033 PMC3798510

[B33] Kim D. Paggi J. M. Park C. Bennett C. Salzberg S. L. (2019). Graph-based genome alignment and genotyping with HISAT2 and HISAT-genotype. *Nat. Biotechnol.* 37 907–915. 10.1038/s41587-019-0201-4 31375807 PMC7605509

[B34] Kitagawa R. Law E. Tang L. Rose A. M. (2002). The Cdc20 homolog, FZY-1, and its interacting protein, IFY-1, are required for proper chromosome segregation in *Caenorhabditis elegans*. *Curr. Biol.* 12 2118–2123. 10.1016/s0960-9822(02)01392-1 12498686

[B35] Lara-Gonzalez P. Kim T. Desai A. (2017). Taming the beast: control of APC/C(Cdc20)-dependent destruction. *Cold Spring Harb. Symp. Quant. Biol.* 82 111–121. 10.1101/sqb.2017.82.033712 29133301 PMC6374126

[B36] Lee C. S. Putnam A. Lu T. He S. Ouyang J. P. T. Seydoux G. (2020). Recruitment of mRNAs to P granules by condensation with intrinsically-disordered proteins. *Elife* 9:e52896. 10.7554/eLife.52896 31975687 PMC7007223

[B37] Lee M. H. Schedl T. (2006). RNA-binding proteins. *WormBook* 18 1–13. 10.1895/wormbook.1.79.1 18050487 PMC4781538

[B38] Lin R. (2003). A gain-of-function mutation in oma-1, a C. elegans gene required for oocyte maturation, results in delayed degradation of maternal proteins and embryonic lethality. *Dev. Biol.* 258 226–239. 10.1016/s0012-1606(03)00119-2 12781695

[B39] Listovsky T. Zor A. Laronne A. Brandeis M. (2000). Cdk1 is essential for mammalian cyclosome/APC regulation. *Exp. Cell Res.* 255 184–191. 10.1006/excr.1999.4788 10694434

[B40] Love M. I. Huber W. Anders S. (2014). Moderated estimation of fold change and dispersion for RNA-seq data with DESeq2. *Genome Biol.* 15:550. 10.1186/s13059-014-0550-8 25516281 PMC4302049

[B41] Marangos P. Verschuren E. W. Chen R. Jackson P. K. Carroll J. (2007). Prophase I arrest and progression to metaphase I in mouse oocytes are controlled by Emi1-dependent regulation of APC(Cdh1). *J. Cell Biol.* 176 65–75. 10.1083/jcb.200607070 17190794 PMC2063628

[B42] Matova N. Cooley L. (2001). Comparative aspects of animal oogenesis. *Dev. Biol.* 231 291–320. 10.1006/dbio.2000.0120 11237461

[B43] Minevich G. Park D. S. Blankenberg D. Poole R. J. Hobert O. (2012). CloudMap: a cloud-based pipeline for analysis of mutant genome sequences. *Genetics* 192 1249–1269. 10.1534/genetics.112.144204 23051646 PMC3512137

[B44] Moore M. J. (2005). From birth to death: the complex lives of eukaryotic mRNAs. *Science* 309 1514–1518. 10.1126/science.1111443 16141059

[B45] Newport J. Kirschner M. (1982). A major developmental transition in early Xenopus embryos: I. characterization and timing of cellular changes at the midblastula stage. *Cell* 30 675–686. 10.1016/0092-8674(82)90272-0 6183003

[B46] Noble S. L. Allen B. L. Goh L. K. Nordick K. Evans T. C. (2008). Maternal mRNAs are regulated by diverse P body-related mRNP granules during early *Caenorhabditis elegans* development. *J. Cell Biol.* 182 559–572. 10.1083/jcb.200802128 18695046 PMC2500140

[B47] Oh D. Houston D. W. (2017). RNA localization in the vertebrate oocyte: establishment of oocyte polarity and localized mRNA assemblages. *Results Probl. Cell Differ.* 63 189–208. 10.1007/978-3-319-60855-6_9 28779319 PMC6538070

[B48] Oldenbroek M. Robertson S. M. Guven-Ozkan T. Spike C. Greenstein D. Lin R. (2013). Regulation of maternal Wnt mRNA translation in *C. elegans* embryos. *Development* 140 4614–4623. 10.1242/dev.096313 24131629 PMC3817945

[B49] Parker D. M. Winkenbach L. P. Boyson S. Saxton M. N. Daidone C. Al-Mazaydeh Z. A. (2020). mRNA localization is linked to translation regulation in the *Caenorhabditis elegans* germ lineage. *Development* 147:dev186817. 10.1242/dev.186817 32541012 PMC7358130

[B50] Passmore L. A. (2004). The anaphase-promoting complex (APC): the sum of its parts? *Biochem. Soc. Trans.* 32 (Pt 5) 724–727. 10.1042/BST0320724 15493998

[B51] Passmore L. A. Booth C. R. Venien-Bryan C. Ludtke S. J. Fioretto C. Johnson L. N. (2005). Structural analysis of the anaphase-promoting complex reveals multiple active sites and insights into polyubiquitylation. *Mol. Cell* 20 855–866. 10.1016/j.molcel.2005.11.003 16364911

[B52] Pesin J. A. Orr-Weaver T. L. (2007). Developmental role and regulation of cortex, a meiosis-specific anaphase-promoting complex/cyclosome activator. *PLoS Genet.* 3:e202. 10.1371/journal.pgen.0030202 18020708 PMC2077894

[B53] Peters J. M. (2002). The anaphase-promoting complex: proteolysis in mitosis and beyond. *Mol. Cell* 9 931–943. 10.1016/s1097-2765(02)00540-3 12049731

[B54] Peters J. M. (2006). The anaphase promoting complex/cyclosome: a machine designed to destroy. *Nat. Rev. Mol. Cell Biol.* 7 644–656. 10.1038/nrm1988 16896351

[B55] Pines J. (2006). Mitosis: a matter of getting rid of the right protein at the right time. *Trends Cell Biol.* 16 55–63. 10.1016/j.tcb.2005.11.006 16337124

[B56] Pines J. (2011). Cubism and the cell cycle: the many faces of the APC/C. *Nat. Rev. Mol. Cell Biol.* 12 427–438. 10.1038/nrm3132 21633387

[B57] Pintard L. Willems A. Peter M. (2004). Cullin-based ubiquitin ligases: Cul3-BTB complexes join the family. *EMBO J.* 23 1681–1687. 10.1038/sj.emboj.7600186 15071497 PMC394240

[B58] Powell-Coffman J. A. Knight J. Wood W. B. (1996). Onset of *C. elegans* gastrulation is blocked by inhibition of embryonic transcription with an RNA polymerase antisense RNA. *Dev. Biol* 178 472–483. 10.1006/dbio.1996.0232 8812143

[B59] Primorac I. Musacchio A. (2013). Panta rhei: the APC/C at steady state. *J. Cell Biol.* 201 177–189. 10.1083/jcb.201301130 23589490 PMC3628523

[B60] Radford H. E. Meijer H. A. de Moor C. H. (2008). Translational control by cytoplasmic polyadenylation in *Xenopus oocytes*. *Biochim. Biophys. Acta* 1779 217–229. 10.1016/j.bbagrm.2008.02.002 18316045 PMC2323027

[B61] Rappleye C. A. Tagawa A. Lyczak R. Bowerman B. Aroian R. V. (2002). The anaphase-promoting complex and separin are required for embryonic anterior-posterior axis formation. *Dev. Cell* 2 195–206. 10.1016/s1534-5807(02)00114-4 11832245

[B62] Robertson S. Lin R. (2015). The maternal-to-zygotic transition in *C. elegans*. *Curr. Top. Dev. Biol.* 113 1–42. 10.1016/bs.ctdb.2015.06.001 26358869

[B63] Rosario R. Childs A. J. Anderson R. A. (2017). RNA-binding proteins in human oogenesis: balancing differentiation and self-renewal in the female fetal germline. *Stem Cell Res.* 21 193–201. 10.1016/j.scr.2017.04.008 28434825 PMC5446320

[B64] Rose L. Gonczy P. (2014). Polarity establishment, asymmetric division and segregation of fate determinants in early *C. elegans* embryos. *WormBook* 10 1–43. 10.1895/wormbook.1.30.2 25548889

[B65] Satyanarayana A. Kaldis P. (2009). Mammalian cell-cycle regulation: several Cdks, numerous cyclins and diverse compensatory mechanisms. *Oncogene* 28 2925–2939. 10.1038/onc.2009.170 19561645

[B66] Schier A. F. (2007). The maternal-zygotic transition: death and birth of RNAs. *Science* 316 406–407. 10.1126/science.1140693 17446392

[B67] Shakes D. C. Sadler P. L. Schumacher J. M. Abdolrasulnia M. Golden A. (2003). Developmental defects observed in hypomorphic anaphase-promoting complex mutants are linked to cell cycle abnormalities. *Development* 130 1605–1620. 10.1242/dev.00385 12620985 PMC1805483

[B68] Shimada M. Yokosawa H. Kawahara H. (2006). OMA-1 is a P granules-associated protein that is required for germline specification in *Caenorhabditis elegans* embryos. *Genes Cells* 11 383–396. 10.1111/j.1365-2443.2006.00945.x 16611242

[B69] Shirayama M. Soto M. C. Ishidate T. Kim S. Nakamura K. Bei Y. (2006). The conserved kinases CDK-1, GSK-3, KIN-19, and MBK-2 promote OMA-1 destruction to regulate the oocyte-to-embryo transition in *C. elegans*. *Curr. Biol.* 16 47–55. 10.1016/j.cub.2005.11.070 16343905

[B70] Spike C. A. Coetzee D. Nishi Y. Guven-Ozkan T. Oldenbroek M. Yamamoto I. (2014). Translational control of the oogenic program by components of OMA ribonucleoprotein particles in *Caenorhabditis elegans*. *Genetics* 198 1513–1533. 10.1534/genetics.114.168823 25261697 PMC4256769

[B71] Swan A. Schupbach T. (2007). The Cdc20 (Fzy)/Cdh1-related protein, Cort, cooperates with Fzy in cyclin destruction and anaphase progression in meiosis I and II in *Drosophila*. *Development* 134 891–899. 10.1242/dev.02784 17251266 PMC2787194

[B72] Tenenhaus C. Schubert C. Seydoux G. (1998). Genetic requirements for PIE-1 localization and inhibition of gene expression in the embryonic germ lineage of *Caenorhabditis elegans*. *Dev. Biol.* 200 212–224. 10.1006/dbio.1998.8940 9705228

[B73] Tenenhaus C. Subramaniam K. Dunn M. A. Seydoux G. (2001). PIE-1 is a bifunctional protein that regulates maternal and zygotic gene expression in the embryonic germ line of *Caenorhabditis elegans*. *Genes Dev.* 15 1031–1040. 10.1101/gad.876201 11316796 PMC312670

[B74] Timmons L. Fire A. (1998). Specific interference by ingested dsRNA. *Nature* 395:854. 10.1038/27579 9804418

[B75] Tsukamoto T. Gearhart M. D. Spike C. A. Huelgas-Morales G. Mews M. Boag P. R. (2017). LIN-41 and OMA ribonucleoprotein complexes mediate a translational repression-to-activation switch controlling oocyte meiotic maturation and the oocyte-to-embryo transition in *Caenorhabditis elegans*. *Genetics* 206 2007–2039. 10.1534/genetics.117.203174 28576864 PMC5560804

[B76] Uzunova K. Dye B. T. Schutz H. Ladurner R. Petzold G. Toyoda Y. (2012). APC15 mediates CDC20 autoubiquitylation by APC/C(MCC) and disassembly of the mitotic checkpoint complex. *Nat. Struct. Mol. Biol.* 19 1116–1123. 10.1038/nsmb.2412 23007861 PMC3498062

[B77] Verlhac M. H. Terret M. E. Pintard L. (2010). Control of the oocyte-to-embryo transition by the ubiquitin-proteolytic system in mouse and *C. elegans*. *Curr. Opin. Cell Biol.* 22 758–763. 10.1016/j.ceb.2010.09.003 20943362

[B78] Wang J. T. Seydoux G. (2013). Germ cell specification. *Adv. Exp. Med. Biol.* 757 17–39. 10.1007/978-1-4614-4015-4_2 22872473 PMC4966528

[B79] Whitfield Z. J. Chisholm J. Hawley R. S. Orr-Weaver T. L. (2013). A meiosis-specific form of the APC/C promotes the oocyte-to-embryo transition by decreasing levels of the Polo kinase inhibitor matrimony. *PLoS Biol.* 11:e1001648. 10.1371/journal.pbio.1001648 24019759 PMC3760765

[B80] Yang H. Vallandingham J. Shiu P. Li H. Hunter C. P. Mak H. Y. (2014). The DEAD box helicase RDE-12 promotes amplification of RNAi in cytoplasmic foci in C. elegans. *Curr. Biol.* 24 832–838. 10.1016/j.cub.2014.01.008 24684930 PMC4226741

[B81] Yeong F. M. (2004). Anaphase-promoting complex in *Caenorhabditis elegans*. *Mol. Cell Biol.* 24 2215–2225. 10.1128/MCB.24.6.2215-2225.2004 14993261 PMC355850

