# Supplementary figures and images for: The APC/CFZY–1/Cdc20 Complex Coordinates With OMA-1 to Regulate the Oocyte-to-Embryo Transition in Caenorhabditis elegans
Source: Front Cell Dev Biol. 2021 Oct 15;9:749654. doi: 10.3389/fcell.2021.749654 (PMC8554129; doi:10.3389/fcell.2021.749654)

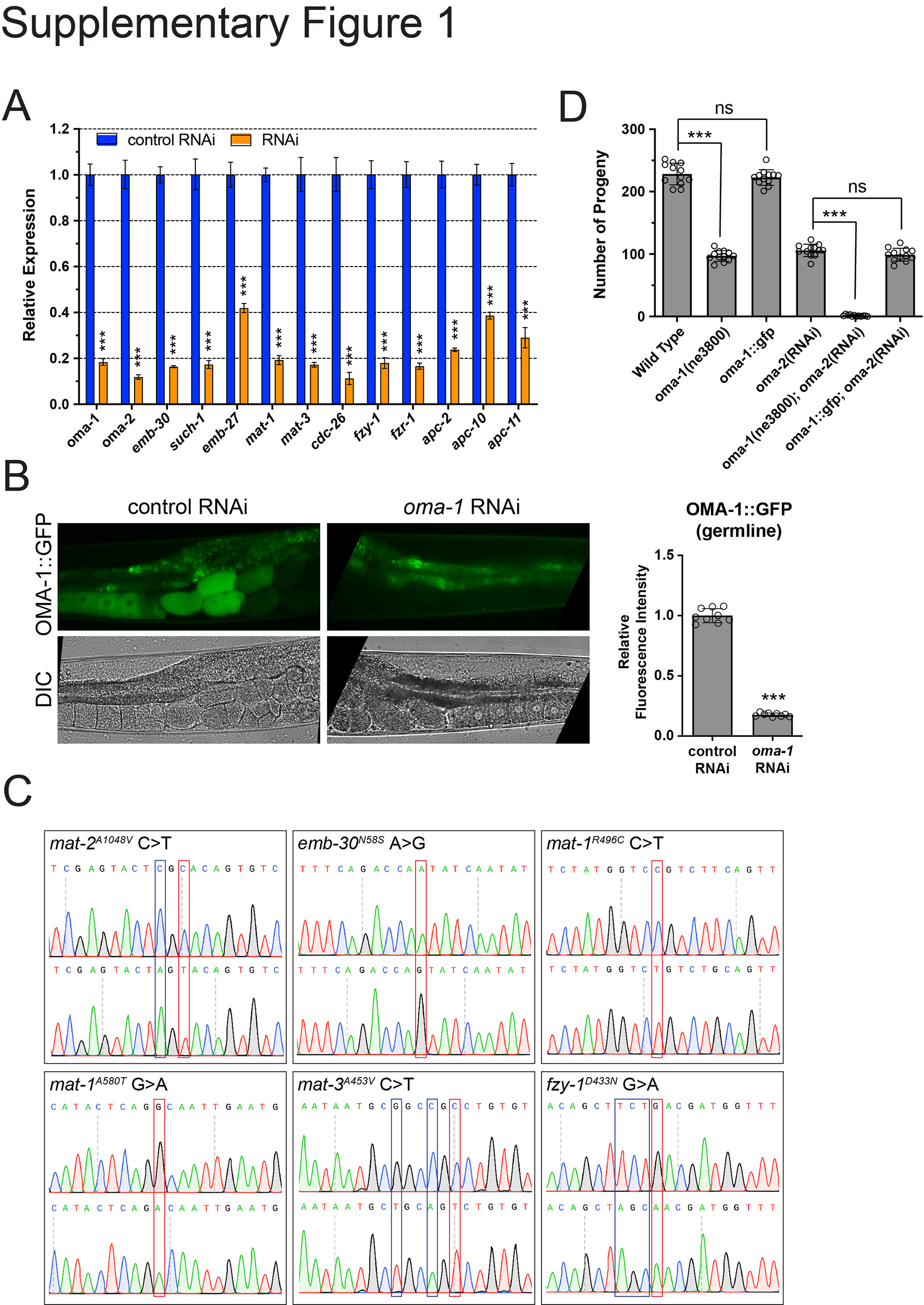

Supplement: Supplementary Figure 1 — (A) Quantitative real-time PCR measurements of expression levels of indicated APC/C genes in RNAi treated strains. The results are presented as the average ± S.D. ∗∗∗p < 0.001; ns: not significant. The p-values were calculated by 2-way ANOVA followed by Dunnett’s multiple comparisons. (B) Fluorescent micrographs show OMA-1::GFP in germlines in control and oma-1 RNAi treated strains to demonstrate oma-1 RNAi efficiency. Quantification of overall OMA-1::GFP fluorescence in germlines is presented as the average ± S.D. with individual values plotted. ∗∗∗p < 0.001. The p-values were calculated by student’s t-test. N = 8–12. (C) Validation of mutations generated by CRISPR/Cas9 gene editing system by Sanger Sequencing. Wild type sequences are shown at the top and mutated sequences are shown at the bottom for each mutation. Red box, missense mutations causing desired amino acid residue change; blue box, silent mutations for genotyping. (D) Brood size of oma-1(ne3800) and oma-1:gfp strains in wild type and oma-2 RNAi background. The results are presented as the average ± S.D. with individual values plotted. ∗∗∗p < 0.001; ns: not significant. The p-values were calculated by 1-way ANOVA followed by Dunnett’s multiple comparisons. N = 10–15. [file Image_1.jpeg]
