# Supplementary material for: The APC/CFZY–1/Cdc20 Complex Coordinates With OMA-1 to Regulate the Oocyte-to-Embryo Transition in Caenorhabditis elegans
Source: Front Cell Dev Biol. 2021 Oct 15;9:749654. doi: 10.3389/fcell.2021.749654 (PMC8554129; doi:10.3389/fcell.2021.749654)
Supplement: Supplementary file 2 [file Data_Sheet_1.PDF]

The APC/CFZY-1/Cdc20 complex coordinates with OMA-1 to regulate the oocyte-to-embryo transition in *C. elegans*

Table S1. *C. elegans* strains used in this study

Strain	Genotype	Method
TX20	*oma-1(zu405) IV*	CGC
EGD175	*pie-1::gfp III;mex-5(F294N,F339N) IV*	CGC
HCC21	*gfp::mex-3 II*	CGC
DG4213	*gfp::meg-1 X*	CGC
DG4222	*gfp::pos-1 V*	CGC
DZ209	*oma-1::gfp IV*	CRISPR/Cas9
DZ471	*oma-1(zu405)::gfp IV*	CRISPR/Cas9
DZ211	*fzy-1(D433N) II*	CRISPR/Cas9
DZ613	*mat-1(A580T) I*	CRISPR/Cas9
DZ616	*mat-1(R496C) I*	CRISPR/Cas9
DZ624	*mat-2(A1048V) II*	CRISPR/Cas9
DZ626	*mat-3(A453V) III*	CRISPR/Cas9
DZ692	*emb-30(N58S) III*	OUTCROSS
DZ228	*fzy-1(D433N) II;oma-1(zu405) IV*	CROSS
DZ688	*mat-1(A580T) I;oma-1(zu405) IV*	CROSS
DZ689	*mat-1(R496C) I;oma-1(zu405) IV*	CROSS
DZ690	*mat-2(A1048V) II;oma-1(zu405) IV*	CROSS
DZ691	*mat-3(A453V) III;oma-1(zu405) IV*	CROSS
DZ230	*fzy-1(D433N) II;oma-1::gfp IV*	CROSS
DZ481	*fzy-1(D433N) II;oma-1(zu405)::gfp IV*	CROSS
DZ306	*oma-1::gfp mRuby::pgl-1 IV*	CROSS
DZ307	*fzy-1(D433N) II;oma-1::gfp mRuby::pgl-1 IV*	CROSS
DZ529	*fzy-1(D433N) II;oma-1(zu405)::gfp mRuby::pgl-1 I*	CROSS
DZ530	*oma-1(zu405)::gfp mRuby::pgl-1 IV*	CROSS
DZ402	*pie-1::gfp III*	OUTCROSS
DZ300	*fzy-1(D433N) II;pie-1::gfp III*	CROSS
DZ360	*pie-1::gfp III;oma-1(zu405) IV*	CROSS
DZ361	*fzy-1(D433N) II;pie-1::gfp III;oma-1(zu405) IV*	CROSS
DZ403	*gfp::mex-3 II;oma-1(zu405) IV*	CROSS
DZ469	*fzy-1(D433N) gfp::mex-3 II*	CRISPR/Cas9
DZ548	*fzy-1(D433N) gfp::mex-3 II;oma-1(zu405) IV*	CROSS
DZ676	*fzy-1(D433N) II;gfp::meg-1 X*	CROSS
DZ677	*gfp::meg-1 X;oma-1(zu405) IV*	CROSS
DZ678	*fzy-1(D433N) II;gfp::meg-1 X;oma-1(zu405) IV*	CROSS
DZ679	*fzy-1(D433N) II;gfp::pos-1 V*	CROSS
DZ680	*gfp::pos-1 V;oma-1(zu405) IV*	CROSS
DZ681	*fzy-1(D433N) II;gfp::pos-1 V;oma-1(zu405) IV*	CROSS
DZ701	*mat-1(A580T) I;pie-1::gfp III;*	CROSS
DZ702	*mat-1(A580T) I;pie-1::gfp III;oma-1(zu405) IV*	CROSS
DZ703	*mat-2(A1048V) II;pie-1::gfp III;*	CROSS
DZ704	*mat-2(A1048V) II;pie-1::gfp III; oma-1(zu405) IV*	CROSS

Table S2. Oligonucleotide sequences strains used in this study

Purpose	Oligo name	Oligo sequence
CRISPR/sgRNA	oma-1::gfp sgRNA	TGACCAAGCATCTCAATTTG
	oma-1(zu405) sgRNA	ATCAACTCCAGATGAGCCAG
	fzy-1(D433N) sgRNA	ATGCTAACAGCTTCTGACGA
	mat-1(R496C) sgRNA	AGCTCTTCCCAACTGAAGA
	mat-1(A580T) sgRNA	ATTACAAAGACAGCATACTC
	mat-2(A1048V) sgRNA	CGCACAGTGTCTAGTTATG
	mat-3(A453V) sgRNA	CTTCTGTACGAAACACAGG
CRISPR donor construct	oma-1::gfp donar F1	AAAACGACGGCCAGTGAATTCTACTCGTGGTCCAAGATATGAGCT
	oma-1::gfp donar R1	CGGCGGCCGCCCAtAAGTTGAGATGCTTGGTCATATTTTCGG
	oma-1::gfp donar F2	GAACTTATGGGCGGCCGCGCGGCCATGAGTAAA
	oma-1::gfp donar R2	CGCGGCGGCCCTATTTGTATAGTTACATCCATG
	oma-1::gfp donar F3	ATACAAATAGGGCCGCCGCCGCCTGAACAACTTC
	oma-1::gfp donar R3	CTTGCATGCCTGCAGGTCGACAAATTTGAAAATTGAATCGATCAAA
	oma-1(zu405) donar	TTAGAAATGTTTGCCAGGCCATCAACTCTAGATGAGCCAGCTGCTAAATTGCCACTAGGACCAACTCCTG
	fzy-1(D433N) donar	CGTCCGTACTCTGAAATGCTAACAGCTAGCAACGATGGTTTCCTGCGTATTTACCGATT
	mat-1(R496C) donar	TATCATTCTTAACACGTCTATGGTCTGTCTGCAGTTGGGAAGAGCTTGCTTCGAGC
	mat-1(A580T) donar	GTGCCGCTGGAAATTGCTTCTCACTGCAGAGACAGCATACTCAGACAATTGAATGTATGGAGAGGGCTAT
	mat-2(A1048V) donar	CTGATTCCATGTATTCTCGAGTACTAGTACAGTGTCTAGTTATGTGGGATTCG
	mat-3(A453V) donar	ACACGAATTCATGGAAATGAAGAATAATGCTGCAGTCTGTGTTTCGTACAGAAGAGCTATTG
qPCR	actin-3 qPCR F	GGCCCAATCCAAGAGAGGTATCC
	actin-3 qPCR R	GGGCAACACGAAGCTCATTGTA
	ptr-2 qPCR F	TTCCGACCTACACAATCATGG
	ptr-2 qPCR R	TCGCTGATGCAAGGATATCTG
	mex-3 qPCR F	GATAGGCGTGCAAAATGTCAC
	mex-3 qPCR R	TGATGTAGGTGTTGGTCTTGG
	htp-1 qPCR F	GCGTTTTCGGCTGTTCTTAG
	htp-1 qPCR R	ATCCCCAGCATTTCGAAGTAG
	cif-1 qPCR F	AGCTTCCAGTTTTCGCCTAC
	cif-1 qPCR R	TTGAGAGTGGAGCAGATTTCG
	zwl-1 qPCR F	GTAAGATTGCAACGGAGAATCG
	zwl-1 qPCR R	GCTTATCTCCCCATCCCAAATC
	cdc-25.1 qPCR F	CCCGAGGAAGATGTGGATTTTAG
	cdc-25.1 qPCR R	CGCCCTGTTGGTGTATCATAG
	ima-2 qPCR F	GACTCTGTATACTCTTATGCTCACTG
	ima-2 qPCR R	TGCTCCGATTGACTTTCCTG
	oma-2 qPCR F	ACCCCAGTTAGCATTCGTG
	oma-2 qPCR R	TGGACGAGTTGCCATATTCAG
	pgl-1 qPCR F	TCCAACGAAATCAGTAGCTCTG
	pgl-1 qPCR R	CAGGAAGAGGCATTGGAGTAG
	nos-2 qPCR F	CGTAAGAAATGCGACAAGCTC
	nos-2 qPCR R	TCTCGGCTGAAATCCTCATTG
	puf-6 qPCR F	GTTGAATCGTGATGCGTTGG
	puf-6 qPCR R	CCATCCAGAGTAGAGAAGCAG
	pos-1 qPCR F	GTGTGATGCTTACAAACGCAG
	pos-1 qPCR R	AACCGTCTTGTACTTGGGATG
	skpt-1 qPCR F	CTACGGATTGGACCCTAATATGG
	skpt-1 qPCR R	TTGAAGTACTGACGGTTGACC
	apx-1 qPCR F	AGACTGTGTTAACGAGACGC
	apx-1 qPCR R	GAACAGATTCCACCGTTTGC
	cdk-1 qPCR F	AAGATGAATACCTTCCGCTCG
	cdk-1 qPCR R	CTTGATTGCTCCATTGTTGTCG
	elli-1 qPCR F	AGAACCCACTGGATAGCAAAG
	elli-1 qPCR R	CTCGCATAGTGTCATTCCTACG
	daz-1 qPCR F	ACCGACTTCTCTTCCATCAAC
	daz-1 qPCR R	GTTCACGCAACTCAGTTTCTG
	meg-1 qPCR F	CCAAAGCCTCAAACTCATCG
	meg-1 qPCR R	TTATCAAGACTGGCAGCGG
	cyb-3 qPCR F	GGATGGTTGAGATTCAGGAGAC
	cyb-3 qPCR R	CAATGAAGATGGCGACACATG
	cpg-1 qPCR F	CCGCTTATGTTGCCAGATATCC
	cpg-1 qPCR R	ACTTCAGGTTTGTATCCGACG
	plk-1 qPCR F	ACAACTACACCATTCCGTCG
	plk-1 qPCR R	GAAGTCTAGTCGGCATGAATCC
	oma-1 qPCR F	CCAACTCCTGTTAGTACTCGTG
	oma-1 qPCR R	TGCCATATTCCAAGCGTCTAG
	gld-1 qPCR F	CTAATTCGCTTTTGTCGCCAG
	gld-1 qPCR R	CCTCGATTGGTTCTGTAGCTG
